# Development of double-positive thymocytes at single-cell resolution

**DOI:** 10.1186/s13073-021-00861-7

**Published:** 2021-03-26

**Authors:** Young Li, Kun Li, Lianbang Zhu, Bin Li, Dandan Zong, Pengfei Cai, Chen Jiang, Pengcheng Du, Jun Lin, Kun Qu

**Affiliations:** 1 Department of oncology, The First Affiliated Hospital of USTC, Division of Molecular Medicine, Hefei National Laboratory for Physical Sciences at Microscale, School of Basic Medicine, Division of Life Sciences and Medicine, University of Science and Technology of China, Hefei, 230021 Anhui China; 2 The CAS Key Laboratory of Innate Immunity and Chronic Disease, CAS Center for Excellence in Molecular Cell Sciences, University of Science and Technology of China, Hefei, 230021 Anhui China; 3 School of Data Science, University of Science and Technology of China, Hefei, 230027 Anhui China

**Keywords:** Single-cell sequencing, Thymocyte, Cell cycle, Thymic selection

## Abstract

**Background:**

T cells generated from thymopoiesis are essential for the immune system, and recent single-cell studies have contributed to our understanding of the development of thymocytes at the genetic and epigenetic levels. However, the development of double-positive (DP) T cells, which comprise the majority of thymocytes, has not been well investigated.

**Methods:**

We applied single-cell sequencing to mouse thymocytes and analyzed the transcriptome data using Seurat. By applying unsupervised clustering, we defined thymocyte subtypes and validated DP cell subtypes by flow cytometry. We classified the cell cycle phases of each cell according to expression of cell cycle phase-specific genes. For immune synapse detection, we used immunofluorescent staining and ImageStream-based flow cytometry. We studied and integrated human thymocyte data to verify the conservation of our findings and also performed cross-species comparisons to examine species-specific gene regulation.

**Results:**

We classified blast, rearrangement, and selection subtypes of DP thymocytes and used the surface markers CD2 and Ly6d to identify these subtypes by flow cytometry. Based on this new classification, we found that the proliferation of blast DP cells is quite different from that of double-positive cells and other cell types, which tend to exit the cell cycle after a single round. At the DP cell selection stage, we observed that CD8-associated immune synapses formed between thymocytes, indicating that CD8sp selection occurred among thymocytes themselves. Moreover, cross-species comparison revealed species-specific transcription factors (TFs) that contribute to the transcriptional differences of thymocytes from humans and mice.

**Conclusions:**

Our study classified DP thymocyte subtypes of different developmental stages and provided new insight into the development of DP thymocytes at single-cell resolution, furthering our knowledge of the fundamental immunological process of thymopoiesis.

## Background

The thymus gland is a specialized organ that is highly conserved in vertebrates [1]. In the thymus, most CD4/CD8 double-negative (DN) precursors develop into the αβ-T cell lineage and then go through CD4/CD8 double-positive (DP) and CD4/CD8 single-positive (SP) stages to achieve maturation [2]. While developing into DP cells, thymocytes undergo rapid and extensive proliferation, which generates a burst of DP cells and supplies a large pool of T cell clones [3, 4]. When DP cells become quiescent from expansion, rearrangement of the TCRα chain is initiated to form mature TCRs. Then DP thymocytes are subjected to positive and negative selection, which depend on TCR interaction with the peptide/major histocompatibility complex (MHC) complex (pMHC). In general, the TCRα chain is continuously rearranged until a MHC-restricted TCR αβ heterodimer is achieved [5, 6]. Although DP cells are strongly sensitive to TCR stimulation [7, 8], most DP cells appear to be neglected with no appropriate pMHC signals in their lifespan and undergo programmed cell death. Only DP thymocytes bearing TCRs that interact with self-peptide/MHC complexes of low affinity will acquire MHC restriction and differentiate into CD4/CD8 SP cells. A nonself-reactive TCR repertoire is ultimately shaped through negative selection. Thymocytes that survive the thymopoiesis process become naïve T cells and migrate out of the thymus.

Traditional studies have mainly focused on bulk-level analyses and have largely depended on known markers of relevant developmental stages. In recent years, single-cell sequencing technology has offered researchers the opportunity to explore scientific issues from a much wider and deeper perspective. For example, Kernfeld et al. and Zeng et al. reported a comprehensive depiction of the development of the fetal thymus in mice and humans, respectively [9, 10]. Lavaert et al. studied the single-cell transcriptional dynamics of human postnatal thymus seeding progenitors [11], and Zhou et al. revealed the regulatory gene expression dynamics leading to lineage commitment in DN thymocytes [12]. These studies have helped us to understand the development of early thymocytes from a variety of aspects. However, the DP cells that account for approximately 80% of the proportion in the mature thymus were either not present among [9, 10] or selectively excluded from [11, 12] the target cell types used in these studies. Recently, Park et al. presented a comprehensive landscape of human thymocyte development [13]. In addition, Le et al. described the lineage specification trajectories and commitment spectrum of human thymocytes [14]. Nevertheless, the development of DP cells in mice has not been well characterized to date.

In this study, we performed single-cell sequencing of mouse thymocytes and reconstructed the entire developmental trajectory in detail. We classified the main cell types of thymocytes and identified three subtypes in the DP stage. We then developed a flow cytometry gating strategy to partition these DP subtypes at the protein level. Based on unsupervised classification and pseudotime analysis, we found that DP thymocytes undergo an unique mechanism of cell division that is different from that of DN cells and other cell types. During the selection stages, the activity of MHC-I molecule-associated antigen presentation was significantly upregulated, suggesting a process of antigen presentation and recognition between thymocytes. To confirm this hypothesis, we examined immune synapses between thymocytes. Moreover, we carried out cross-species comparisons and identified species-specific transcription factors (TFs) that contribute to the transcriptional differences between thymocytes from humans and mice. Together, our study provides new insight into the development of DP thymocytes at single-cell resolution, and these findings help us to better understand this fundamental immunological process.

## Methods

### Study design

The objective of this study was to use scRNA-seq to capture the transcriptomes of αβ-T cells in the thymus to describe important events during αβ-T cell development. Flow cytometry was employed to confirm observations from sequencing datasets to validate expression levels at baseline. Immunofluorescence and imaging flow cytometry were used to assess the existence of CD8-specific selection between thymocytes. Details on the sample collection and processing are described below.

### Mice

C57BL/6 mice were ordered from Beijing Vital River Laboratory Animal Technology and maintained under specific pathogen-free conditions until the experiments were performed. All mouse experiments in this study were reviewed and approved by the Institutional Animal Care and Use Committee of the University of Science and Technology of China.

### Thymocyte isolation

Thymus tissues were harvested from mice aged 6–8 weeks and gently ground in 1 mL of RPMI-1640. Thymocytes in a single-cell suspension were counted after being passed through a 40-μm nylon mesh filter.

### Flow cytometry

For surface marker staining, cells were labeled with fluorescent antibodies at 4 °C for 30 min and washed twice with 1× phosphate-buffered saline (PBS, Sangon, China). For intracellular marker staining, the cells were fixed with 1% paraformaldehyde (PFA, Sangon) at 4 °C for 10 min, washed with 1× perm/wash buffer (BD Bioscience) once, and incubated in 1× perm/wash buffer for 30 min at 4 °C. Next, the cells were labeled with fluorescent antibodies at 4 °C for 30 min and washed twice with 1× perm/wash buffer. After the final wash, the cells were analyzed or sorted using an SH800S cell sorter (Sony).

### Imaging synapses between thymocytes

To maintain the natural attachment between adjacent thymocytes, we gently cut the thymus with surgical scissors in an Eppendorf tube; 1 mL of 1× PBS was added to resuspend the thymocytes, and the thymic debris was allowed to settle. The suspension was pipetted and passed through a 40-μm nylon mesh filter. After counting the cells, approximately 1 × 10^7^ thymocytes were labeled with fluorescent anti-mouse antibodies at 4 °C for 30 min in approximately 100 μL 1× PBS. Then, 30 μL of 4% PFA was directly added and gently mixed, and the cells were fixed at 4 °C for 10 min. The cells were briefly centrifuged at 100×g for 1 min to avoid the formation of a tight cell pellet and resuspended in 200 μL of 1× perm/wash buffer. After staining with 488-labeled phalloidin for 20 min at room temperature, the cells were centrifuged at 100×*g* for 1 min and resuspended in 60 μL of 1× PBS. For ImageStream experiments, cells were directly examined using an Amnis ImageStream Mk II Imaging Flow Cytometer (Luminex). For immunofluorescence experiments, cells were diluted to a proper concentration, seeded on poly-l-lysine-coated slides and observed by confocal microscopy (ZEISS LMS 880).

### Cell proliferation staining

DPbla/re cells were sorted using the surface markers CD45^+^CD4^+^CD8^+^CD2^low^, and the total thymocyte population and DPbla/re cells were separately labeled with UltraGreen (AAT Bioquest) cell dye. After culturing in serum-free UltraCulture medium (Lonza) for 24 h, proliferative cells were identified by fluorescence intensity analysis.

### Antibodies

Anti-mouse CD4 (BV421, PE, APC, and Percp-Cy5.5), CD8 (FITC, APC, and PE-Cy7), Ly6d (FITC), CD2 (PE and APC), CD69 (PE), Ki67 (BV421), CD3e (BV421), and H2 (PE) antibodies were purchased from BioLegend. Anti-mouse RORγt (PE) antibodies were obtained from BD Bioscience. All fluorescent antibodies were used according to the user manuals.

### Single-cell sequencing

Single cells were captured in droplet emulsions using a GemCode Single-Cell Instrument (10X Genomics), and scRNA-seq libraries were constructed with the 10X Genomics protocol using a GemCode Single-Cell 3′ Gel Bead and Library V2 Kit. The libraries were sequenced using a HiSeq X-10 Sequencing System (Illumina). scATAC-seq experiments were performed as previously described [15].

### Data processing

Sequences were aligned using Cell Ranger version 1.3.1 from 10X Genomics with default parameters. The GRCm38.p5 assembly was used as the reference genome, and ribosomal RNA, mitochondrial RNA (Mt-RNA), and pseudogenes were removed. Cells with fewer than 500 detected genes and more than 4500 detected genes for which total mitochondrial gene expression exceeded 40% were removed. Genes that were expressed in fewer than three cells were also removed. Quantile normalization was performed using qnorm in R.

### Identification of cell types and subtypes

Standard procedures for filtering, variable gene selection, dimensionality reduction, clustering, and identifying marker genes were performed using the Seurat package version 2.3.0.

1. FindVariableGenes (x.low.cutoff = 0.025, x.high.cutoff = 3, y.cutoff = 0.5).
2. FindClusters (reduction.type = “pca”, dims.use = 1:20, resolution=2, print.output=0).
3. RunTSNE (dims.use = 1:20, do.fast = TRUE).
4. FindAllMarkers (only.pos = TRUE, min.pct = 0.5, thresh.use=0.5).

### Merging Tabula Muris data

We downloaded raw thymocyte single-cell RNA-seq data from Tabula Muris [16]. Sequencing data were aligned using Cell Ranger version 1.3.1 from 10X Genomics with default parameters. The genome build used was GRCm38.p5 without pseudogenes, ribosomal genes, and mitochondrial genes. Then, we used Seurat (V 3.1.4) to integrate two datasets for analysis with the following key parameters.

1. FilterCells (nFeature_RNA > 1000, nFeature_RNA < 5000 and percent.mt < 10).
2. FindVariableFeatures (nfeatures = 2000).
3. FindIntegrationAnchors (dims = 1:40), IntegrateData (dims.use = 1:20).
4. RunPCA (npcs = 40), FindClusters (resolution = 1).

The robustness of clustering was tested by Seurat analysis under 25 different conditions with combinations of five resolution values (Res = 0.8, 0.9, 1, 1.1, 1.2) and five values for the number of neighbors in the initial graph (*k* = 15, 20, 25, 30, 35). We then calculated the consistency of clustering for each cell pair by their co-occurrence count across the 25 parameter settings.

### Developmental trajectory and cell order analysis

After cell filtering, data were prepared for visualization and population balance analysis (PBA) [17] by constructing a *k*-nearest neighbor (kNN) graph, in which cells correspond to graph nodes and edges connect cells to their nearest neighbors. The kNN graphs were visualized using a force-directed layout with a custom interactive software interface called SPRING and default parameters. The cell order was predicted by PBA [17], which calculates a scalar “potential” for each cell that is analogous to a distance, or pseudotime, from an undifferentiated source and a vector of fate probabilities that indicates the distance to fate branch points. For a self-renewing system, the sum of all cells satisfies the constraint ∑_*i*_ *Ri* = 0. We assigned negative values to *R* for the ten cells with the highest expression of CD4sps and CD8sps marker genes. We assigned an SP value to all remaining cells, and the value was chosen to enforce the steady-state condition ∑_*i*_ *Ri* = 0. The cell order was scaled according to the stage of cell development.

### Identifying dynamically varying genes

For each gene, a sliding window (*n* = 20 cells) across the cell ordering was used to identify windows with the maximum and minimum average expression, as previously described [18]. Transition points between stages were defined using the frequency of gene inflection points and patterns of PBA-predicted fate probabilities. However, owing to the continuous nature of transcriptional states, the locations of these transitions should be considered approximate. The inflection point density is the number of genes that turn on or off at a given point on the trajectory. For each gene, inflection points were identified at the points with maximally increasing or decreasing expression, as follows. First, the trajectory of each dynamically varying gene was smoothed using Gaussian smoothing with a width *σ* = 1% of the total trajectory. The gene expression derivative for gene *k*, denoted dk, was then computed. Inflection points were identified as the points with the maximum or minimum derivatives for each gene. To exclude maxima or minima resulting from relatively small fluctuations in gene expression, only appreciably large extrema were retained for further analysis. Specifically, a point with a derivative for gene *k*, max (dk), was kept only if

$$ \max\ \left(\mathrm{dk}\right)/\mathrm{median}\ \left(\mathrm{dk}\right)>Q $$

We chose the threshold *Q* = 3 for this study and then plotted the density of these inflection points over the cell ordering axis. Regions with large-scale changes in gene expression have a high density of inflection points, whereas relatively stable states are characterized by low density [17].

### Enrichment of TFs using SCENIC

Transcription factors from scRNA-seq data were identified using SCENIC [19] with the default parameters. An enrichment score less than the threshold was defined as no enrichment (0), and nCellsAssigned TFs less than 10% and greater than 90% were removed. The average of each subgroup was obtained based on the previous clustering results, and TFs with large differences (> 0.4) between subgroups were identified.

### Mapping the relationship between TFs and global changes in genes

Time-specific TFs and the possible regulatory relationship between TF and genes were obtained from the SCENIC results. Next, TF genes with high correlations were screened out (correlation > 0.4) based on the correlation between TF and gene expression. These TF genes were assigned to different stages according to the TF and gene expression patterns. Circos was then used to map genes regulated by TFs.

### scATAC-seq analysis

We used the general mapping, alignment, peak calling, and motif searching procedures to process the scATAC-seq data from APEC [15] and ATAC-pipe [20]. We also implemented a Python script in ATAC-pipe to trim adapters in the raw data (in paired-end FASTQ files for each single-cell sample). APEC utilizes BOWTIE2 to map trimmed sequencing data to the corresponding genome index and PICARD for sorting, duplicate removal, and fragment length counting of the aligned data. The pipeline calls peaks from the merged file of all cells using MACS2 and ranks and filters out low-quality peaks based on the false discovery rate (*Q*-value). The genomic locations of the peaks were annotated by HOMER, and motifs were searched using FIMO. APEC calculates the number of fragments and the percent of reads mapped to the transcriptional start site (TSS) region (±2000 bp) in each cell and filters out high-quality cells. Finally, we obtained 1933 cells with 600 accessons [15] (peak group with a similar pattern). TF enrichment in each cell was obtained for downstream analysis.

### scRNA-seq and scATAC-seq integration analysis

We merged the genes from scRNA-seq data into gene groups corresponding to accessions based on the genes corresponding to the peak in the accessions above. We then integrated the scRNA-seq and scATAC-seq (in the form of cell × accession) data according to the accessions. The mutual nearest neighbor was used to find the anchor and weight of the two data points on the nearest 100 canonical correlation spaces (60 canonical correlation spaces were used when analyzing the integrated data). The t-SNE and PBA cell order from scRNA-seq data were assigned to each cell for the scATAC-seq data according to the anchor and weight. Finally, the motif enrichment information from scATAC-seq was assigned to each scRNA-seq cell based on the average of the 10 cells of the PBA order. The cell type information from scRNA-seq was assigned to scATAC-seq.

### Cell cycle analysis

We defined genes from the cell cycle Gene Ontology (GO) category in the Mouse Genome Informatics (MGI) database as genes associated with the cell cycle. The average of all cell cycle-related genes was taken as the cell cycle score for each cell. Genes with periodic expression correlating with the cell cycle [18] were used to generate a cell cycle phase score for each cell, after which a phase score was calculated for each phase (G1/S, S, G2/M, M, and M/G1) by averaging expression traces for the genes specific to that phase. Finally, the *z* score of each phase in each cell was calculated, and cells with a *z* score greater than 1 (one standard deviation) were classified into each phase. If the *z* score of a cell in each phase was less than 1, the cell was not considered to be in the cell cycle. The status was divided into different phases (G1/S, S, G2/M, M, M/G1, and not cell cycle) for each cell. Read counts were normalized using qnorm, and numpy.polyfit (deg = 10) was used to fit the data.

### Removing cell cycle-related genes and reclustering analysis

All genes related to the cell cycle (genes from the cell cycle GO term in the MGI) were removed from the data, and then reclustering was performed as previously described (resolution = 1.9). The overlap ratio of the four subpopulations (DPblas and the original subpopulations) after the removal of cell cycle-related genes was determined.

Reclustering of DPblas was also performed in accordance with the previous parameters (resolution = 1).

### Cell cycle phase analysis of the Tabula Muris thymus, erythroid and neuron data sets

The cell cycle phase analysis method used was the same as previously described, and the default parameters in Seurat and PBA were applied to calculate the cell cluster and cell order of the Tabula Muris thymus (Seurat resolution = 2) and neuron (Seurat resolution = 0.2) datasets. We used GSE109774 [16] for the Tabula Muris thymus dataset. We used GSE89754 [17] for the erythroid dataset and GSE93593 [21] for the neuron dataset. The erythroid PBA cell order was based on the results of the original paper.

### Cell cycle phase analysis of the human thymus data sets

The cell cycle phase analysis method employed was the same as previously described, and in Seurat (V 3.1.4), integrated methods (min.cells = 3,min.features = 500, PC = 40, resolution = 1.2) and PBA were applied to calculate the cell cluster and cell order of the 24-year-old human thymus E-MTAB-8581 [13] dataset.

### Cell cycle phase analysis of the early T cell data sets

The cell cycle phase analysis method was the same as previously described. Seurat (V 3.1.4), integrated methods (min.cells = 3,min.features = 500, PC = 40, resolution = 1.2) and PBA were used to calculate the cell cluster and cell order of the early T cell dataset GSE130812 [12].

### Human mouse cross-species comparison analysis

We used Seurat (V 3.1.4) to integrate the mouse and human single-cell thymocyte datasets. The parameters used for the analysis were as follows:

1. FilterCells (nFeature_RNA > 1000, nFeature_RNA < 5000 and percent.mt < 10).
2. FindVariableFeatures (nfeatures = 2000).
3. FindIntegrationAnchors (dims = 1:20), IntegrateData (dims.use = 1:20).
4. RunPCA (npcs = 20), FindClusters (resolution = 1).

Genes that were differentially expressed between the species were identified with two steps: (1) genes that were expressed in one species but not the other (expressed: nCells> 20, not expressed: nCells < 3); (2) for each stage, marker genes (fold change > 0.2, background expression < 1) for human and mouse data were combined, and if the list was larger than 400, only the top 400 significant genes were included. Then, differentially expressed genes across species were identified (fold change > 0.25) for all developmental stages. Genes in either (1) or (2) were regarded as differentially expressed genes between humans and mice.

Cells were divided into 100 bins along the developmental pseudotime trajectory, and smoothed gene expression values were calculated with the numpy.polyfit (*n* = 10) option.

A TF list was obtained from The Human Transcription Factors database [22], while the transcriptomes for human samples were obtained from different datasets (see “Availability of data and materials”). Genes regulated by each TF were based on gene set enrichment analysis (GSEA) [23]. The significance of whether a TF regulates cross-species differential genes was calculated by Fisher’s exact test.

ChIP-seq data for Gata1 were obtained from Cistrome Data Browser [24] with the accession numbers GSM912907, GSM867158, and GSM867159. RNA-seq data for the human and mouse thymus were obtained from ENCODE [25] under accession numbers GSM1220578, GSM1220591, GSM1220592, GSM1220593, GSM1220599, GSM1220601, GSM1010944, GSE78390, GSM970852, GSE93469, and GSE90183. All data were normalized by sequencing depth, and differential analysis was performed with a *t*-test. Genes with a fold change > 1 and *p* value < 0.01 were regarded as significantly differentially expressed.

### Statistical analysis

The detailed statistical methods and parameters used in this study are described above. The original analysis codes and scripts can be accessed at https://github.com/QuKunLab/T-cell-development [26].

## Results

### Single-cell transcriptome profile depicts αβ-T cell development in the thymus

**Fig. 1:**
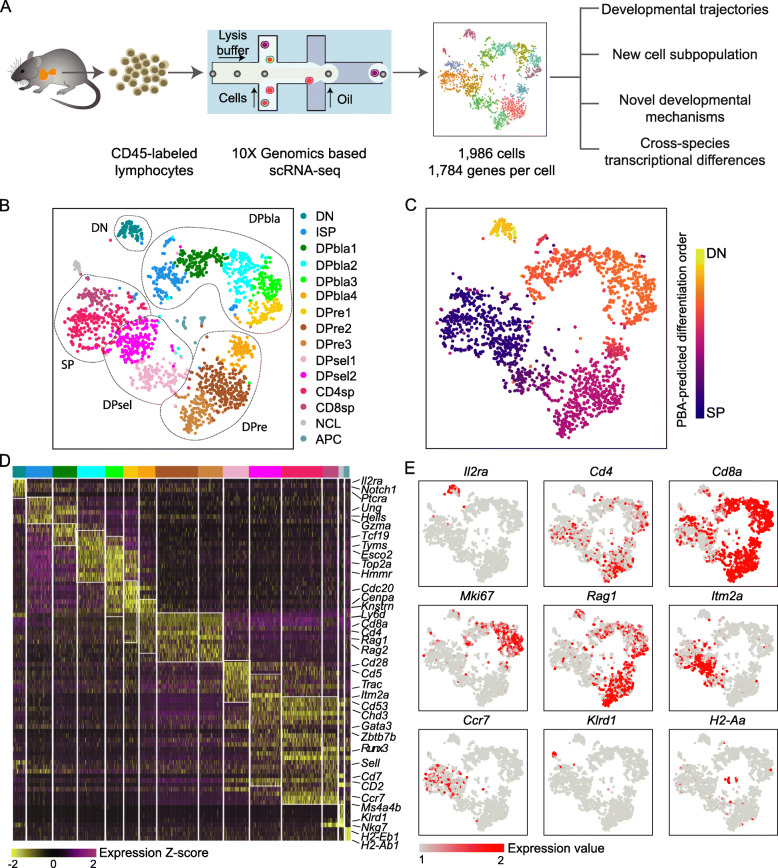
Single-cell transcriptome map of thymocytes. **a** Schematic of procedures for the extraction, sequencing, and single-cell analysis of thymocytes. **b** Two-dimensional representation of cells via t-SNE, as colored by cluster identity; each dot represents one cell. t-SNE was performed after quality control. **c** Two-dimensional representation of cells via tSNE, as colored by the PBA-predicted differentiation order (see “Methods”); each dot represents one cell. **d** Heat map of cluster marker genes (color-coded by clusters), with representative genes labeled (right). Columns denote cells; rows denote genes. **e** *Il2ra, Cd4, Cd8a, Mki67, Rag1, Itm2a, Ccr7, Klrd1* and *H2-Aa* marker genes projected onto t-SNE plots. Color bar, normalized expression value

We profiled lymphocytes in the mouse thymus with a droplet-based single-cell RNA sequencing (scRNA-seq) platform and obtained a total of 2004 cells. Of these, 1986 single cells with an average of 1784 detected genes per cell passed the quality control (see “Methods”) and were used for further analysis (Fig. 1a, Additional file 1: Figure S1A, B). Unsupervised analysis was performed with Seurat [27], and the cells were clustered into 15 subgroups (Fig. 1b). We divided the cells into 7 developmental stages of thymocytes according to the expression of marker genes: DN progenitors (*Il2ra*^+^), immature SP thymocytes (ISPs, *Cd4*^−^ *Cd8*^+^ *Mki67*^+^), DP blasts (DPblas; *Cd4*^+^ *Cd8*^+^ *Mki67*^+^), DP thymocytes undergoing rearrangement (DPres; *Cd4*^+^ *Cd8*^+^ *Rag1*^high^), DP cells under selection (DPsels; *Cd4*^+^ *Cd8*^+^ *Itm2a*^+^), and CD4/CD8 SP thymocytes (CD4sps and CD8sps). Cells in different stages were aggregated in a two-dimensional *t*-distributed stochastic neighbor embedding (t-SNE) plot that outlined the developmental trajectory of αβ-T cells (Fig. 1b).

To verify the developmental stages of the thymocytes we observed, we constructed a pseudotime trajectory using an unsupervised SPRING algorithm and PBA [17], and the predicted timeline was consistent with the clustering results (Additional file 1: Figure S1C, Fig. 1c). Briefly, CD25 (*Il2ra*) represents the most DN subset, and β-selection commits most DN cells to an αβ-T cell fate. When progressing to the ISP stage, *Cd8* expression is turned on, and the thymocytes proliferate rapidly into the DPbla stage with the onset of *Cd4* expression, upregulating cell cycle-associated genes such as Ki-67 (*Mki67*). With the help of *Rag1* and *Rag2* genes, TCRα loci are next rearranged in DPre cells to acquire a mature TCR signal for positive selection in the DPsel stage. Finally, CD4sp and CD8sp cells that survive negative selection will migrate out of the thymus via chemotaxis signals (Fig. 1d, e). Notably, this classification is quite similar to the thymocyte subpopulations in the human thymus [13].

In addition to 13 thymocyte clusters, a group of antigen-presenting cells (APCs) and a group of recently identified nonconventional lymphocytes (NCLs) [9] were defined (Fig. 1d, Additional file 1: Figure S1D). Consistently, many stage-specific markers of thymocyte development were highly expressed in the coordinated clusters (Fig. 1e). To confirm our single-cell partition results, we reanalyzed the thymocyte profile from the Tabula Muris dataset [16] and detected 1364 thymocytes; then, we integrated them with our data and obtained 3320 cells passing quality control. As expected, the clustering results were nearly identical (Additional file 1: Figure S2A, B). Moreover, thymocytes from both datasets were distributed proportionally across clusters with the same markers (Additional file 1: Figure S2C, D). We also compared our single-cell clusters with bulk-sorted thymocyte subpopulations [28] and observed good correlation between the corresponding subtypes of thymocytes (Additional file 1: Figure S2E). To evaluate whether the clustering of the thymocytes was stable, we performed Seurat clustering on the integrated data under 25 conditions with 5 resolutions (Res = 0.8, 0.9, 1.0, 1.1, 1.2) and 5 nearest neighbor values (*K* = 15, 20, 25, 30, 35) (Additional file 1: Figure S3A) and calculated the consistency of the cluster assignment for each cell. We found that pairs of cells that clustered together in the original analyses consistently clustered together across parameter settings (Additional file 1: Figure S3B), which suggests that the clustering results are quite robust.

### Single-cell ATAC-seq revealed new transcription factors involved in thymocyte development

**Fig. 2:**
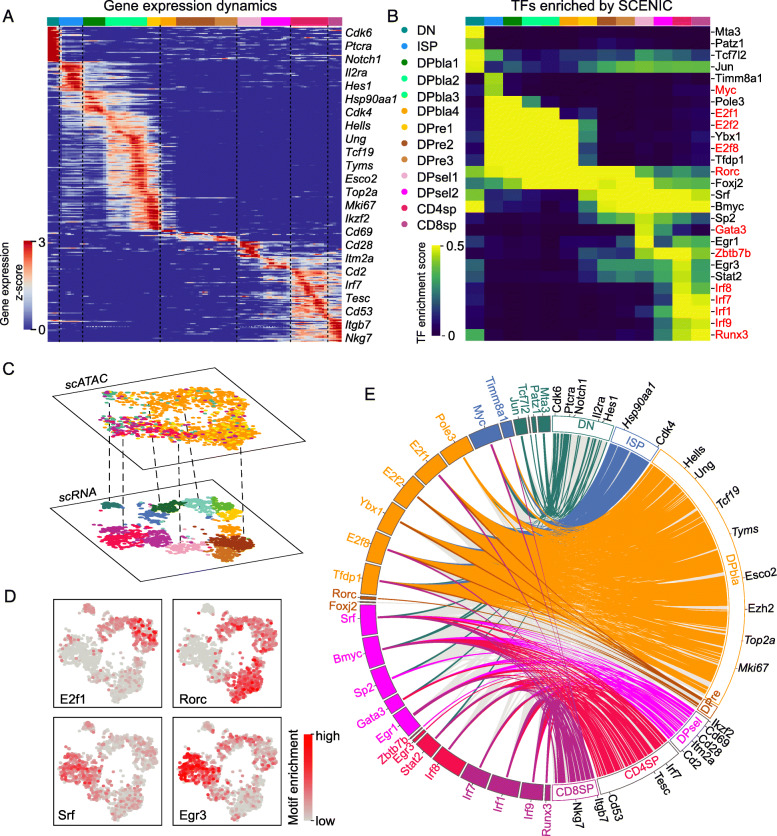
Single-cell ATAC-seq revealed new transcription factors involved in thymocyte development. **a** Heat map of dynamically changing genes during thymocyte development. Cells (columns) are ordered from DN to SP, as predicted by PBA. Genes (rows) are ordered by smoothed peak expression using a Gaussian kernel. **b** Dynamic TF enrichment during thymocyte development, as predicted by SCENIC. Clusters are as labeled in Fig. 1b. The color bar represents the average of the TF enrichment score for each stage. TFs in red were reported to be essential for the development of thymocytes in previous studies. **c** scATAC-seq data were mapped onto scRNA-seq data (see “Methods”). **d** Enrichment of TFs (E2f1, Rorc, Srf and Egr3) during thymocyte development (see “Methods”) in scATAC data. The color bar represents the deviation in TFs. Each dot represents one cell. **e** Circos plot describing the regulation of genes by TFs. The left side shows the TFs enriched at each stage (colors correspond to DN, ISP, DPbla, DPre, DPsel, CD4sp, CD8sp), as shown in Fig. 2b. The right side shows genes differentially expressed at each stage, as in Fig. 2a; some marker genes at each stage are marked outside the circle. The line from left to right indicates that the TFs regulate corresponding genes. The gray link indicates that the correlation between TFs and genes is less than 0.4

The development of thymocytes is driven by the interplay of multiple TFs. However, a comprehensive depiction of TF regulatory dynamics during thymocyte development is lacking. As we constructed the pseudotime development trajectory from the single-cell snapshot of thymocytes (Fig. 1c), we next investigated the dynamics of gene expression during thymocyte development based on the pseudocoordinate of each cell (Fig. 2a). We reconstructed the dynamics of enriched TFs using single-cell regulatory network interference and clustering (SCENIC) [19]. In addition to the essential TFs previously reported, such as Myc, Rorc, Gata3, Zbtb7b and Runx3 [29], novel TFs, such as Srf, were found to be candidate TFs that regulate thymocyte maturation (Fig. 2b). Egr3 is reported to be essential in the proliferation of thymocytes at the transition of DN to DP [30]. Interestingly, we found that Egr3 might also function at the DPsel and SP stages (Fig. 2b).

To validate enrichment of TFs in each stage, we used a single-cell assay for transposase-accessible chromatin using sequencing (scATAC-seq) of thymocytes (Additional file 1: Figure S4A) and anchored the single cells from chromatin accessibility profiles onto those from scRNA-seq (Fig. 2c, Additional file 1: Figure S4B). We measured TF motif enrichment at each developmental stage according to the chromatin-open status of thymocytes and observed TF motif enrichment patterns similar to those of the SCENIC predictions (Additional file 1: Figure S4C). For example, the Srf and Erg3 motifs were highly enriched in DPsels and SP subtypes (Fig. 2d), suggesting that these TFs function in the development of thymocytes. We also mapped scATAC-seq data onto the integrated data and found similar TF enrichment patterns (Additional file 1: Figure S4D-F). Next, we constructed a regulatory network of enriched TFs for each developmental stage according to the coexpression pattern between TFs and marker genes considering the binding motifs in promoter regions (Additional file 1: Figure S5). A Circos plot showed that these stage-specific TFs control expression of marker genes at their stages and helped thymocytes to transition to the next stage (Fig. 2e), illustrating the programmed development progress of thymocytes under the regulation of TFs.

### Ly6d and CD2 can serve as new markers to partition DP subtypes

**Fig. 3:**
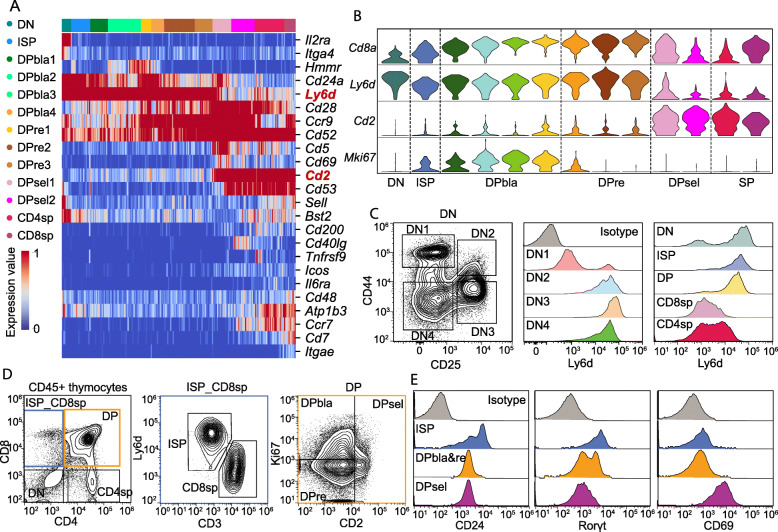
Ly6d and CD2 can serve as new markers to gate DP subtypes. **a** Heat map of selected cell surface marker genes (color-coded by clusters). Columns denote cells; rows denote genes. **b** Violin plots showing normalized expression levels of selected marker genes that changed during the course of T cell development (Cd8a, Ly6d, Cd2 and Mki67). **c** Flow cytometry measurements of Ly6d expression in thymocyte subpopulations. **d** Flow cytometry gating strategy for identifying ISPs and the DP subset. **e** Flow cytometry measurement of CD24, CD3, Rorγt and CD69 expression in ISPs and the DP subtype

DP cells account for approximately 80% of thymocytes and undergo essential processes of proliferation, rearrangement and selection. However, the subclassification of DP cells remains unclear due to a lack of specific markers for distinguishing thymocytes undergoing different developmental stages. In this study, we classified DP cells into three subtypes at the transcriptome level according to the RNA expression pattern and relative biofunction of marker genes: DPbla, DPre, and DPsel. To identify specific markers, we explored differentially expressed cell surface marker genes across thymocyte subtypes (Fig. 3a) and observed that *Ly6d* was highly expressed in thymocytes but dramatically decreased in DPsels and SP cells. In contrast, expression of *Cd2* was relatively low before the DPsel stage but was extensively elevated in DPsels and SP cells (Fig. 3a, b). When integrating with Tabula Muris data, we observed very similar expression patterns of *Ly6d* and *CD2* across thymocyte subtypes (Additional file 1: Figure S6). These results are consistent with previously reported transcriptional dynamics at the bulk level [28]. To verify whether surface marker expression is consistent with RNA expression, we performed flow cytometry analysis for Ly6d. As expected, Ly6d expression increased from the DN2 stage and remained high until the SP stage (Fig. 3c).

Ly6d is reported to identify the branch point of B cell and T cell development [31], but its behavior in T cell development has not been described. CD2 is essential for thymic selection [32]. Thus, Ly6d and CD2 are potential markers to distinguish the developmental stages of DP thymocytes, and we performed flow cytometry analysis to assess whether Ly6d and CD2 may serve as new markers to partition DP subtypes. Together with Ki67 and CD3, we successfully identified ISPs and three DP subtypes: ISPs, CD4^−^CD8^+^Ly6d^+^CD3^−^; DPblas, CD4^+^CD8^+^CD2^low^Ki67^+^; DPres, CD4^+^CD8^+^Ly6d^hi^CD2^low^Ki67^−^; and DPsels, CD4^+^CD8^+^CD2^hi^ (Fig. 3d). To verify this gating strategy, we also examined several known stage-specific markers, such as CD24, Rorγt, and CD69. CD24 was highly expressed on early thymocytes [33] and downregulated from DPblas and DPres. Rorγt, a DP marker [34], was differentially expressed on DP subtypes and downregulated on DPsels. CD69, a marker for DP cell selection [35], was upregulated on DPsels. These protein expression dynamics confirmed that the DP subtypes we defined were at different developmental stages (Fig. 3e).

### DPblas differentiate into DPres during the cell cycle

**Fig. 4:**
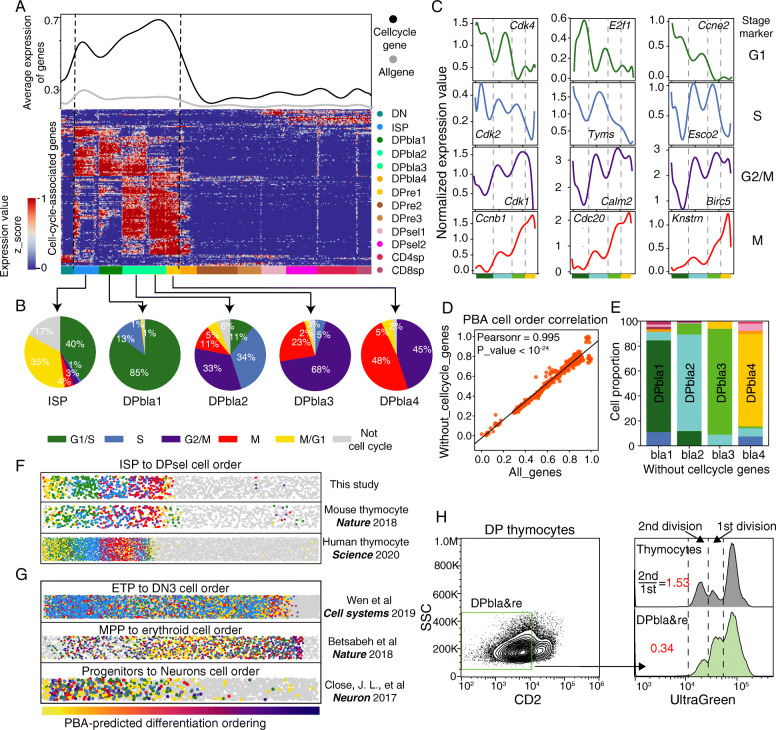
Unique division mechanism of DPbla thymocytes. **a** Top: Mean expression of cell cycle-associated genes (black) and all genes (gray) in each cell. Bottom: Heat map of cell cycle-associated gene expression in each cell. Genes (rows) were sorted by unsupervised clustering. Cells were ordered from DN to SP. **b** Single cells were assigned to different stages of the cell cycle (G1/S, S, G2/M, M, and M/G1) according to their expression of cell cycle stage-specific genes (see “Methods”). The percentage of cells located in each cell cycle stage is shown in pie charts for ISPs and DPblas (DPbla1, DPbla2, DPbla3 and DPbla4). **c** Expression of representative cell cycle genes in corresponding cell cycle stages. **d** Pearson correlation between the cell orders predicted by PBA before and after the removal of cell cycle-related genes. **e** The overlap ratio of the four DPbla subpopulations before and after the removal of cell cycle-related genes (see “Methods”). **f**, **g** Cell cycle stages of single cells were assigned as in Fig. 4a and are shown on a strip plot. Each dot represents one cell. Cells were ordered by PBA prediction. **f** Thymocytes were ordered from DN to SP for our data, Tabula Muris data and human thymus data. **g** Cells were ordered from early T cell progenitor (ETP) cells to DN3 cells for DN thymocytes, from multipotent progenitor (MPP) cells to erythroid cells for erythroid data and from progenitors to neurons for neuron development data. The color scheme indicates that the cell cycle periods were the same as in Fig. 4b, and gray indicates that the cell was not in the cell cycle. **h** The indicated cells (total thymocytes, DPblas and DPres) were sorted and stained with UltraGreen and then cultured in vitro for 24 h. Decayed fluorescence peaks represent dividing cells. The ratio of cells that divided once and twice was calculated as shown

When committed to the αβ-lineage, thymocytes undergo rapid proliferation and transition to DP cells. Consistently, Mki67 was highly expressed on ISPs and DPblas (Fig. 1e). To investigate the proliferation process in detail, we quantified the average expression level of all cell cycle-associated genes during the developmental process and observed strong proliferation of ISPs and DPblas (Fig. 4a). Interestingly, we noticed that the expression patterns of cell cycle-associated genes in ISPs and DPbla1–4 were quite different, indicating different proliferation statuses. Therefore, we classified the cell cycle phases (G1/S, S, G2/M, M or M/G1) of all thymocytes based on phase-specific gene expression [18] (see “Methods”). We found that 40% of ISPs were in M/G1 phase, indicating strong continuous cell cycle proliferation. However, when cells entered the DPbla stage, the M/G1 phase was rarely observed (Fig. 4b), indicating that most DPblas exited the cell cycle after proliferation. Consistent with this, the four cell cycle phases appeared to be distributed in chronological order along the developmental trajectory of DPblas, whereby most DPbla1s were in G1/S phase, DPbla2s were in S and G2/M phases, and DPbla3s and DPbla4s mainly stayed in G2/M and M phases (Fig. 4b). The expression dynamics of cell cycle phase-specific genes also coordinated with the chronological order of the cell cycle phases in DPblas (Fig. 4c).

The observed correlation of cell cycle phases and predicted DPbla stages might be caused by the following: (1) the single-cell clustering and pseudotime prediction analysis were dominated by cell cycle genes or (2) DPbla cells differentiated into DPres during the cell cycle and tended to exit the cell cycle after division. Thus, we excluded all cell cycle-associated genes and reanalyzed the cell clustering and pseudotime trajectory with the same parameters (Additional file 1: Figure S7) and found that the chronological order of cells with or without cell cycle-associated genes correlated significantly (Pearson *r* = 0.995, *p* value < 10^− 24^, Fig. 4d). The distributions of cell identities at the four DPbla stages were also very similar (Fig. 4e). These results suggest that DPbla cells differentiate into DPres during the cell cycle and exit the cell cycle after division.

To test this hypothesis, we analyzed publicly available single-cell RNA profiles of mouse and human thymocytes (Additional file 1: Figure S8, 9) [12, 13, 16] in the same manner. Similar to our data, we observed a conserved chronological distribution of cell cycle phases in mouse and human DPblas (Fig. 4f). However, during the proliferation of DN thymocytes, the cell cycle phases seemed not to be sequentially distributed (Fig. 4g, Additional file 1: Figure S10), suggesting different proliferation mechanisms in DN and DP thymocytes. We also assessed proliferation during the development of other cell types, such as erythroid cells [17] and neurons [21] (Additional file 1: Figure S11), though the cell cycle phases were randomly distributed during the proliferation stages in both cell types, suggesting that the chronological distribution of the phases in development may be DPbla specific (Fig. 4f, g).

To validate the tendency of DPblas to exit the cell cycle, we sorted DPblas/DPres, cultured them in vitro, and quantified the proportion of proliferative cells among DPblas/DPres by UltraGreen staining. For DPblas, only 14% of proliferating cells divided more than once compared to 43% in total thymocytes (Fig. 4h), confirming that DPblas tended to exit the cell cycle after division. In addition, we investigated differentially expressed genes between DPbla subgroups and found that genes involved in T cell recombination and selection were enriched at the end of the DPbla stage (Additional file 1: Figure S12). This result suggests that the cell cycle process during the DPbla stage may help in preparing for the initiation of TCRα chain recombination and the positive selection progress, which occurs in DPres.

### Thymocytes serve as APCs for CD8-associated thymic selection

Following the DPbla stage, thymocytes in the DPre stage enter a relatively quiescent phase [30] with little alteration in gene expression (Fig. 2a) to achieve TCRα chain rearrangement. After TCRα chain recombination and TCR maturation are complete, thymocytes are subjected to thymic selection in the DPsel stage. We identified three DPre and two DPsel subtypes. To verify differences among these subtypes, we analyzed differentially expressed genes between the DPre and DPsel subtypes (Additional file 1: Figure S13A, B) and found that these subtypes appear to be in different developmental stages. For example, mitochondrial cytochrome c oxidase-associated genes were enriched in DPre1, suggesting high metabolic activity. In contrast, genes involved in SRP-dependent cotranslational protein targeting the membrane were enriched in DPre2, which might result from the TCR translocation process. Enrichment of the GO term “T cell differentiation” in the DPre3 cluster might indicate maturation of the TCR signal at the late rearrangement stage (Figure S13C). In terms of DPsel subtypes, DPsel1 cells were highly enriched with the GO term “T cell costimulation”, suggesting that these cells undergo an active selection process. The “cytoplasmic translation” GO term was associated with DPsel2 cells, indicating that these cells are preparing for differentiation into the next stage (Figure S13D).

**Fig. 5:**
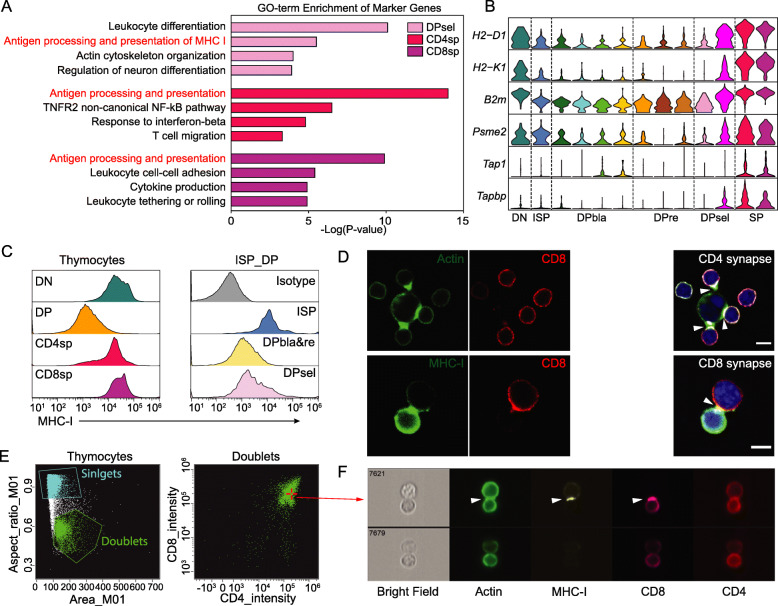
MHC-I antigen presentation occurred between thymocytes. **a** Top enriched GO terms for marker genes of DPsels, CD4sps and CD8sps. GO term enrichment analysis was performed with Metascape. **b** Violin plots showing normalized expression levels of selected marker genes that changed during the course of T cell differentiation (H2-D1, H2-K1, B2 m and Tapbp). **c** Flow cytometry measurements of MHC-I expression in thymocyte subpopulations. **d** Immune synapse detection by confocal microscopy. The observed immune synapses are indicated by white arrows. The color bar represents 5 μm. **e** Gating scheme for thymocyte doublets by ImageStream flow cytometry. **f** The upper panel shows an example of an immune synapse (indicated with white arrows) formed between adjacent thymocytes. The bottom panel shows an example of adjacent thymocytes with no significant interactions

In addition, we observed strong enrichment of the MHC-I antigen presentation process in DPsels, CD4sps, and CD8sps (Fig. 5a). For example, expression of MHC-I, such as *H2-D1*, *H2-K1*, and *B2 m*, which are essential for antigen presentation, was significantly increased in DPsels and SP cells compared to DPre cells (Fig. 5b). Immunoproteasome subunits such as *Psme2* and antigen loading-associated genes such as *Tap1* and *Tapbp* were also upregulated in these stages (Fig. 5b). Furthermore, increased MHC-I antigen presentation activity at selection stages was observed in the integrated mouse and human thymocyte data (Additional file 1: Figures S14, S15). We then performed flow cytometry analysis and verified upregulation of surface MHC-I molecules in the DPsels and SP cells (Fig. 5c). These findings suggest an active MHC-I antigen presentation process in DPsels and SP cells.

CD8-associated positive and negative selection of thymocytes depends on the interaction of CD8, TCR, and the peptide-MHC-I (pMHCI) complex. However, the source of pMHCI has not been well studied. Classically, thymic epithelial cells (TECs) and APCs are considered major contributors. Nevertheless, it has been reported that thymocytes still exhibit normal CD8sp differentiation when pMHCI is expressed only on hematopoietic cells [36]. Moreover, soluble pMHCI can effectively rescue CD8sp differentiation in an MHC-I-deficient fetal thymic organ culture system [37, 38]. These data suggest that CD8sp selection may respond to a wide source of pMHCI, which may include pMHCI on thymocytes themselves. In addition, expression of thymoproteasome subunits, which promote CD8 T cell differentiation [39, 40], was detected in thymocytes (Additional file 1: Figure S16); therefore, these cells are capable of providing a pMHCI signal for CD8-associated thymic selection.

We observed high expression of *Cd2* by DPsels and SPs, which is critical for the formation of immunological synapses (ISs) [32, 41]. Thus, when DPsels and SPs interact intensively with proper pMHC on APCs during the selection process, ISs can be observed between them. To investigate whether selection occurs between thymocytes, we performed immunofluorescence experiments to detect ISs formed between thymocytes and adjacent APCs. We found that DP and CD4sp cells formed ISs with CD4-CD8-APCs in the thymus, indicating active CD4-associated selection between them (Fig. 5d). In addition to CD4-specific ISs, we detected CD8-associated ISs between thymocytes (Fig. 5d). When we further investigated adjacent thymocytes by imaging flow cytometry (Fig. 5e), we also observed clear CD8-associated ISs between adjacent DP and SP cells with CD4 evenly distributed on the surface (Fig. 5f). This finding suggests active CD8-associated selection between thymocytes. These results demonstrate that thymocytes can serve as APCs for CD8-associated thymic selection.

### Species-specific TFs regulate cross-species transcriptional differences during thymocyte development in humans and mice

**Fig. 6:**
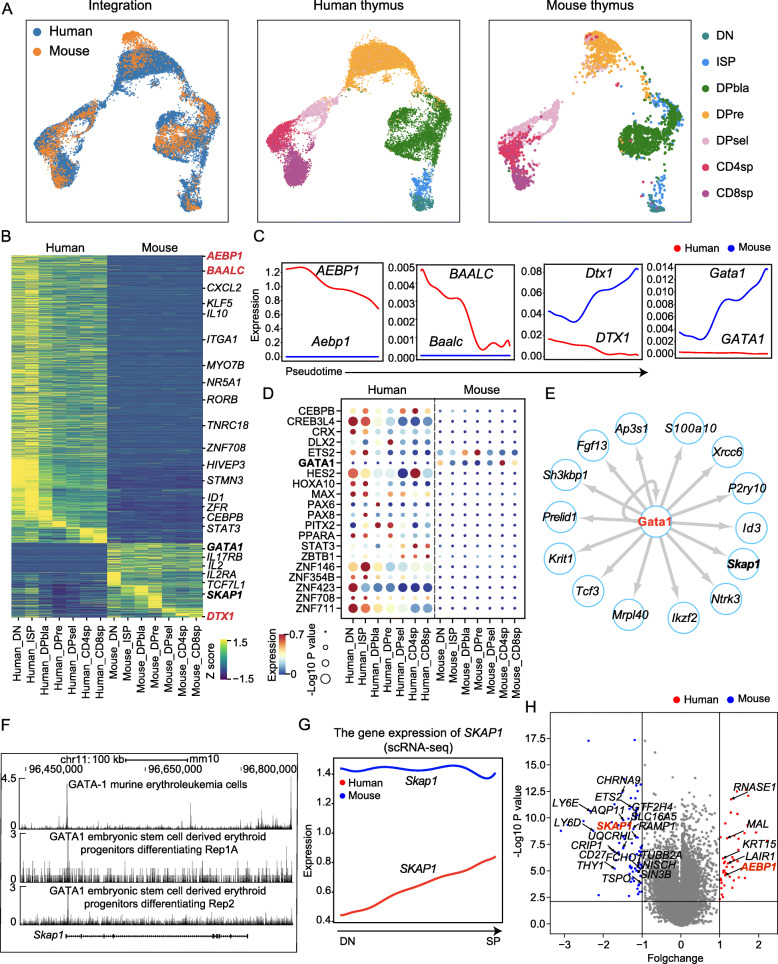
Species-specific transcriptional regulation during thymocyte development between humans and mice. **a** Two-dimensional representation of cells via UMAP, as colored by species (left), cluster identity in humans (middle) and cluster identity in mice (right); each dot represents one cell. **b** A heat map of differentially expressed genes between species, with exemplar genes labeled (right); genes in red were reported to be differentially expressed between these species, and genes in bold are discussed below. **c** Expression of representative genes (AEBP1, BAALC, DTX1 and GATA1) involved in the development of T cells. The value is the smoothed gene expression value. **d** Expression of TFs across stages in humans and mice. The color indicates the average expression value; the circle indicates the −log10 *p* value of TF enrichment. **e** The transcriptional regulatory network of Gata1, which shows the genes that are differentially expressed between species in Fig. 6b. **f** Gata1 ChIP-seq profiles of Skap1 gene loci. The Gata1 ChIP-seq data are from murine erythroleukemia cells (top) and ES (embryonic stem) cell-derived erythroid progenitors (middle and bottom). ChIP-seq signals were obtained from Cistrome Data Browser. **g** Expression of SKAP1/Skap1 in humans and mice during the development of T cells. The value is the smoothed gene expression value. **h** Volcano map of human and mouse thymus data. The absolute value of the fold change > 1 and *p* value < 0.01 are regarded as significant differences; mouse is blue and human red; the label is the gene found in Fig. 6b, and the red ones are known (AEBP1) and predicted (SKAP1) differential genes between humans and mice. RNA-seq data were obtained from ENCODE

To investigate cross-species differences in thymocyte development, we integrated the single-cell transcriptome profiles of adult human and mouse thymocytes. Consistent with a previous report [13], the clusters of human and mouse thymocytes were mixed well (Fig. 6a, Additional file 1: Figure S17A), indicating that the development of thymocytes is conserved between these species. Nonetheless, cross-species transcriptional differences at the single-cell level occur during thymocyte development [13, 14]. Accordingly, we compared the single-cell transcriptomes of human and mouse thymocytes across all clusters and found many differentially expressed homologous genes, including previously reported genes such as *AEBP1*, *BAALC*, and *DTX1* [14] (Fig. 6b, c).

To investigate the regulation of transcriptional differences between humans and mice, we focused on differentially expressed TFs (Figure S17B). To this end, we overlapped their regulated genes annotated by MSigDB [42] with the differentially expressed genes between humans and mice and examined the enrichment to determine whether they might drive cross-species transcriptional differences (Fig. 6d). In humans, genes regulated by differential TFs, such as *CREB3L4, HES2, ZNF146, ZNF423,* and *ZNF711,* were significantly enriched among the genes differentially expressed from mice, indicating that these TFs might contribute to cross-species transcriptional differences in humans (Additional file 1: Figure S17B, C). In mice, the candidate driver TFs detected were Ets2 and Gata1 (Fig. 6d, e, Figure S17D). *Ets2* belongs to the ETS-domain transcription factor family, which is critical for many bioprocesses, including development [43]; *Gata1* is known to be essential for erythroid and megakaryocytic differentiation [44]. To verify whether Gata1 is capable of regulating these differentially expressed genes between humans and mice, we analyzed ChIP-seq data for Gata1 and found that it binds to genomic regions around these differentially expressed genes, such as *Skap1*, *Ap3s1,* and *P2ry10* (Fig. 6f, g, Additional file 1: Figure S17E, F). Moreover, *Skap1* was also among the most differentially expressed genes in the human and mouse thymus (Fig. 6h). Thus, Gata1 might play a role in driving cross-species transcriptional differences between humans and mice.

## Discussion

The proper development of T cells is essential for effective responses against invading pathogens and for tolerance against self-antigens in the adaptive immune system; it is therefore a fundamental subject of investigation. In this study, we surveyed the mouse thymocyte development process at a single-cell resolution and classified subtypes of DP thymocytes that were consistent with those of human thymocytes [13]. Unlike previous classifications of DP cells, for which gating strategies were mainly based on expression of a few well-studied marker genes, we classified cell subtypes via unsupervised analysis of high-throughput single-cell transcriptomes and then confirmed them by flow cytometry, rendering our classification a better representation of the inherent heterogeneity between cell subgroups. In addition to DP cells, DN and SP cell clusters were detected in our data. We classified SP cells into CD4sp and CD8sp clusters. A small proportion of CD4sp cells appeared to express CD8 mRNAs, which made CD4sp and CD8sp cells seemed not to be pure. However, we observed a very robust classification of these two clusters across different analysis parameters, suggesting that the CD4sp and CD8sp clusters we identified are indeed transcriptionally different. The moderate expression of CD8 mRNA in CD4sp cells may be caused by the delayed degradation of CD8 mRNAs. Overall, the relatively low cell number of our dataset limited the power in terms of separating SP clusters, and more experiments, such as cell sorting and single-cell sequencing, are needed to fully determine the cell purity of the CD4sp and CD8sp clusters.

During the transition from DN to DP, thymocytes undergo 6–8 divisions [45] to generate a large pool of DP cells. Consistent with this, we observed a notable proportion of M/G1 thymocytes in the DN and ISP stages, indicating continuous cell cycles. Thus, DN thymocytes are self-renewable and support the persistence and differentiation of thymocytes without any input from bone marrow progenitors for a relatively long time. When entering the DPbla stage, thymocytes invoke a different proliferation pathway and differentiate into DPres during the cell cycle, which appears not to be self-renewable. This may be caused by upregulation of RORγt in DP cells, which suppresses the proliferation of thymocytes [30]. Typically, DP cells comprise the majority (60 to 80%) of thymocytes in the first 4 to 5 decades of life. Afterwards, as a consequence of thymic involution, expansion at the early DP stage seems to be disrupted, and DP cells decline dramatically and irreversibly (to less than 15%) [46]. Decreased thymocyte levels result in a deficit in naïve T cell output to the peripheral T cell pool, strongly contributing to the immune insufficiency of older individuals [47]. Hence, proliferation at the early DP stage is crucial to maintain the homeostasis of thymic T cell development. Our study revealed a unique proliferation behavior of DPblas that lacks self-renewal ability. The unique division mechanism of DP thymocytes may lead to a decline in the DP population during thymic involution.

Thymic selection is essential for T cells to establish central tolerance, which depends on the TCR affinity with the self-peptide-MHC complex. Unlike CD4 T cells restricted to MHC-II, which is expressed on only TECs and APCs, CD8 T cells recognize peptides presented on MHC-I, which is widely expressed on a variety of cells, including thymocytes themselves. Our data suggest that upregulated activity of MHC-I antigen presentation in DPsels and SP thymocytes may contribute to CD8sp T cell selection between thymocytes, as also indicated by previously reported data [36–38]. This phenomenon may also be related to the thymic preference for CD4 T cells over CD8 T cells, which is usually thought to be a consequence of the default CD4 T cell pathway [48] or homeostatic mechanisms [49, 50]. Our study results suggest another possibility of easier access to pMHCI in CD8sp selection, accelerating fate decisions for CD8sp cells. Consistent with this, CD8 T cells usually become reconstituted faster than CD4 T cells after hematopoietic stem cell transplantation [51].

## Conclusions

In summary, this study classified the main thymocyte cell types and identified three subtypes of DP cells at different developmental stages. We revealed that Ly6d and CD2 can be used as surface markers to partition these subtypes at the protein level. Based on the classification of DP thymocytes, we found that DPblas differentiate into DPres during the cell cycle, which is specific to DP thymocyte development compared to DN and other cell types. We also observed that at the thymic selection stage, thymocytes can serve as APCs in CD8-associated selection. Based on cross-species comparison, we found that species-specific TFs contribute to the transcriptional differences between thymocytes from humans and mice. Together, this study provides new insight into the development of DP thymocytes at a single-cell resolution and helps us to better understand this fundamental immunological process.

## Supplementary Information

**Additional file 1: Figure S1.** Data processing workflow and quality control of thymocytes. **Figure S2.** Integrated analysis with data from Tabula Muris. **Figure S3.** Robustness of thymocyte clustering. **Figure S4.** Single-cell ATAC-seq on mouse thymocytes. **Figure S5.** The transcription regulation network of each stage during thymocyte development. **Figure S6.** Ly6d and CD2 serve as new markers to gate DP subtypes in integrated data. **Figure S7.** Single-cell transcriptome clustering and pseudo-time trajectory of thymocytes without cell cycle-related genes. **Figure S8.** Single-cell transcriptome map of thymocytes from Tabula Muris dataset. **Figure S9.** Single-cell transcriptome map of thymocytes from Human data. **FigureS10.** Single-cell transcriptome map of Early T Cell Development. **Figure S11.** Single-cell transcriptome map of neuronal development. **Figure S12.** GO terms enriched in subgroups of DPbla stage. **Figure S13.** The difference in subgroups of DP stage. **Figure S14.** MHC-I antigen presentation occurred between thymocytes in integrated data. **Figure S15.** The expression of MHC-I associated genes in thymocytes. **Figure S16.** The expression of thymoproteasome subunits associated genes in thymocytes. **Figure S17.** Transcriptional regulation differences between human and mouse thymocyte development.

## Data Availability

The raw sequence data reported in this paper have been deposited in the Gene Expression Omnibus (GEO) under accession numbers GSE166715, which is also publicly accessible from https://www.ncbi.nlm.nih.gov/geo/query/acc.cgi?acc=GSE166715 [52]. The processed data are provided on figshare (the thymus single-cell data generated from this study: https://figshare.com/s/1f910888df4afa4ec53b [53], the integrated thymus single-cell data: https://figshare.com/s/db3f03095c74c16cf38d [54]). The original analysis codes and scripts can be accessed at https://github.com/QuKunLab/T-cell-development [26]. The Tabula Muris thymus dataset was downloaded from GSE109774: https://www.ncbi.nlm.nih.gov/geo/query/acc.cgi?acc=GSE109774 [55]. The erythroid dataset was downloaded from GSE89754: https://www.ncbi.nlm.nih.gov/geo/query/acc.cgi?acc=GSE89754 [56]. The neuron dataset was downloaded from GSE93593: https://www.ncbi.nlm.nih.gov/geo/query/acc.cgi?acc=GSE93593 [57]. The 24-year-old human thymus dataset was downloaded from E-MTAB-8581: https://www.ebi.ac.uk/arrayexpress/experiments/E-MTAB-8581/ [58]. A TF list was obtained from The Human Transcription Factors database: http://humantfs.ccbr.utoronto.ca/ [59]. Genes regulated by each TF were based on GSEA: http://www.gsea-msigdb.org/gsea/msigdb/collections.jsp#C3 [60]. ChIP-seq data for Gata1 were obtained from Cistrome Data Browser: http://cistrome.org/db/#/ [61]. RNA-seq data for the human and mouse thymus were obtained from ENCODE under accession numbers GSM1220578, GSM1220591, GSM1220592, GSM1220593, GSM1220599, GSM1220601 (https://www.ncbi.nlm.nih.gov/geo/query/acc.cgi?acc=GSE18927 [62]), GSM1010944 (https://www.ncbi.nlm.nih.gov/geo/query/acc.cgi?acc=GSE16256 [63]), GSE78390 (https://www.ncbi.nlm.nih.gov/geo/query/acc.cgi?acc=GSE78390 [64], GSM970852 (https://www.ncbi.nlm.nih.gov/geo/query/acc.cgi?acc=GSE49847 [65]), GSE93469 (https://www.ncbi.nlm.nih.gov/geo/query/acc.cgi?acc=GSE93469 [66]) and GSE90183 (https://www.ncbi.nlm.nih.gov/geo/query/acc.cgi?acc=GSE90183 [67]). Code related to analyses is available from GitHub (APEC: https://github.com/QuKunLab/APEC [68], Seurat: https://github.com/satijalab/seurat [69]).

## Abbreviations

DN: Double negative
ISP: Immature single positive
DP: Double positive
DPbla: DP blast
DPre: DP thymocytes undergoing rearrangement
DPsel: DP thymocytes undergoing selection
SP: Single positive
APC: Antigen presentation cell
TEC: Thymic epithelial cell
TCR: T cell receptor
pMHC: Peptide/MHC complex
TF: Transcription factor
