# Supplementary material for: Development of double-positive thymocytes at single-cell resolution
Source: Genome Med. 2021 Mar 26;13:49. doi: 10.1186/s13073-021-00861-7 (PMC8004397; doi:10.1186/s13073-021-00861-7)
Supplement: Supplementary file 1 — Additional file 1: Figure S1. Data processing workflow and quality control of thymocytes. Figure S2. Integrated analysis with data from Tabula Muris. Figure S3. Robustness of thymocyte clustering. Figure S4. Single-cell ATAC-seq on mouse thymocytes. Figure S5. The transcription regulation network of each stage during thymocyte development. Figure S6. Ly6d and CD2 serve as new markers to gate DP subtypes in integrated data. Figure S7. Single-cell transcriptome clustering and pseudo-time trajectory of thymocytes without cell cycle-related genes. Figure S8. Single-cell transcriptome map of thymocytes from Tabula Muris dataset. Figure S9. Single-cell transcriptome map of thymocytes from Human data. FigureS10. Single-cell transcriptome map of Early T Cell Development. Figure S11. Single-cell transcriptome map of neuronal development. Figure S12. GO terms enriched in subgroups of DPbla stage. Figure S13. The difference in subgroups of DP stage. Figure S14. MHC-I antigen presentation occurred between thymocytes in integrated data. Figure S15. The expression of MHC-I associated genes in thymocytes. Figure S16. The expression of thymoproteasome subunits associated genes in thymocytes. Figure S17. Transcriptional regulation differences between human and mouse thymocyte development. [file 13073_2021_861_MOESM1_ESM.docx]

**Supplementary figures**


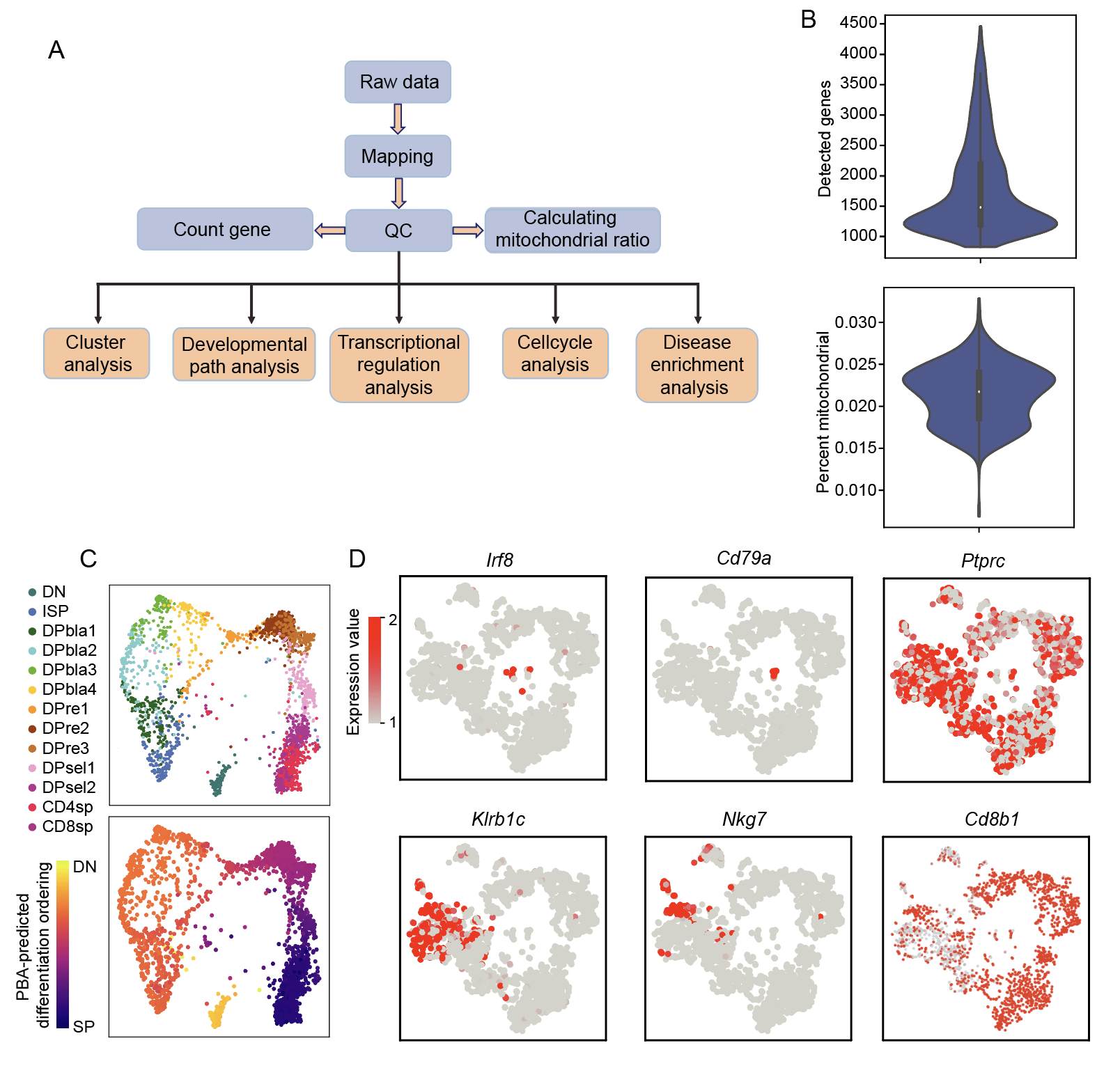


**Fig. S1 Data processing workflow and quality control of thymocytes**

**A:** Data processing workflow.

B: Violin plots of the number of unique genes (top) and the mitochondrial gene fraction (bottom) in 1986 cells.

**C:** Two-dimensional representation of cells via a t-SNE plot, colored by clusters (top) and the PBA-predicted differentiation order (bottom, see Methods); each dot represents one cell.

**D:** Marker genes of APCs and NCLs projected on t-SNE plots. Color bar, normalized expression value.


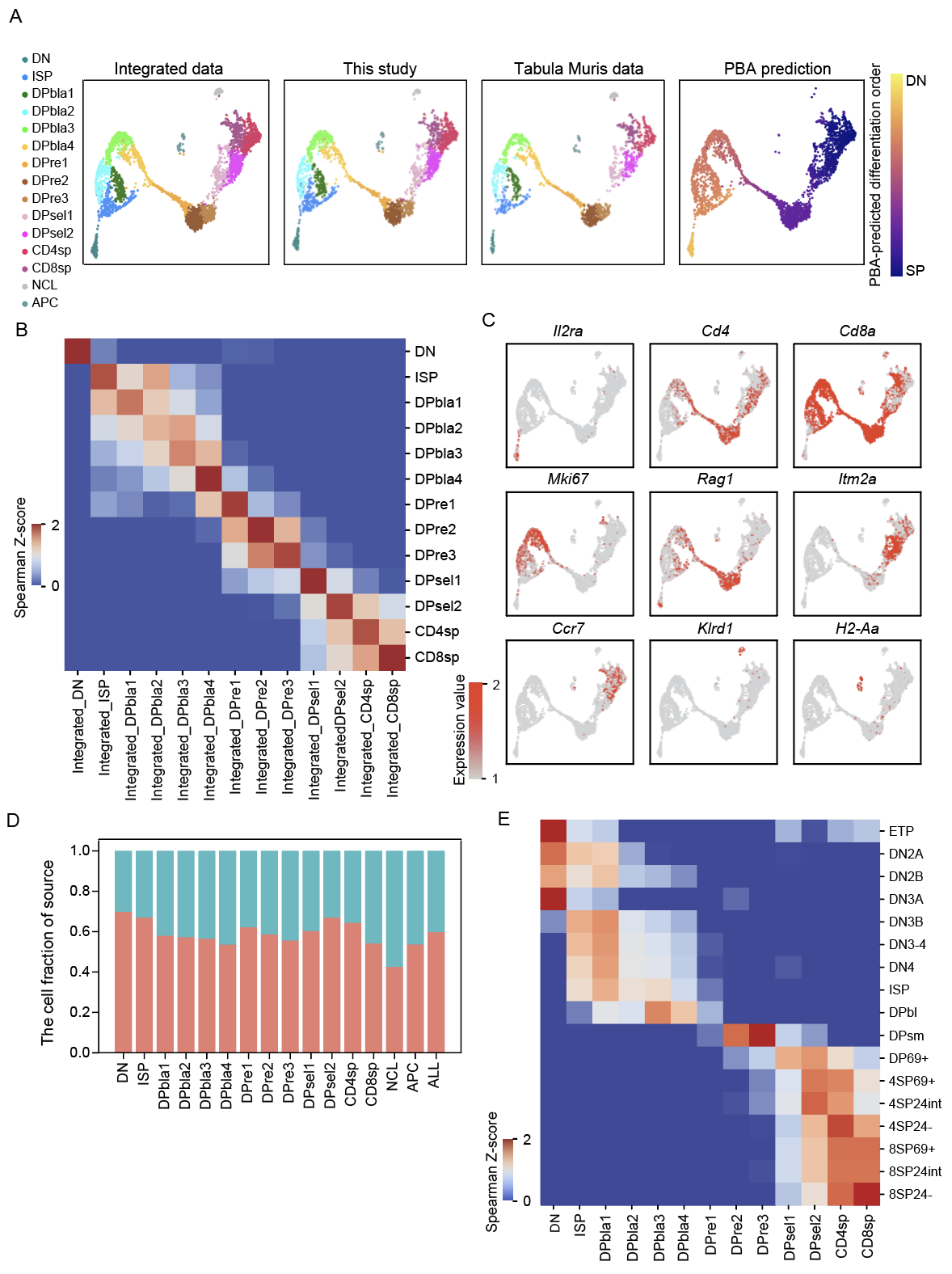


**Fig. S2 Integrated analysis with data from Tabula Muris**

**A:** Two-dimensional representation of cells via UMAP, colored by the cluster identities. First: the integrated data; second: data from this study; third: data from Tabula Muris. The fourth graph shows the PBA-predicted differentiation order from the integrated data. Each dot represents one cell. **B:** Spearman correlation between our data and the integrated data. **C:** *Il2ra*, *Cd4*, *Cd8a*, *Mki67*, *Rag1*, *Itm2a*, *Ccr7*, *Klrd1* and *H2-Aa* marker genes projected on UMAP plots. Color bar, normalized expression value. **D:** Fraction of the data source per cluster. ALL: all cells from this study or Tabula Muris. **E:** Spearman correlation between our data and bulk microarray data.


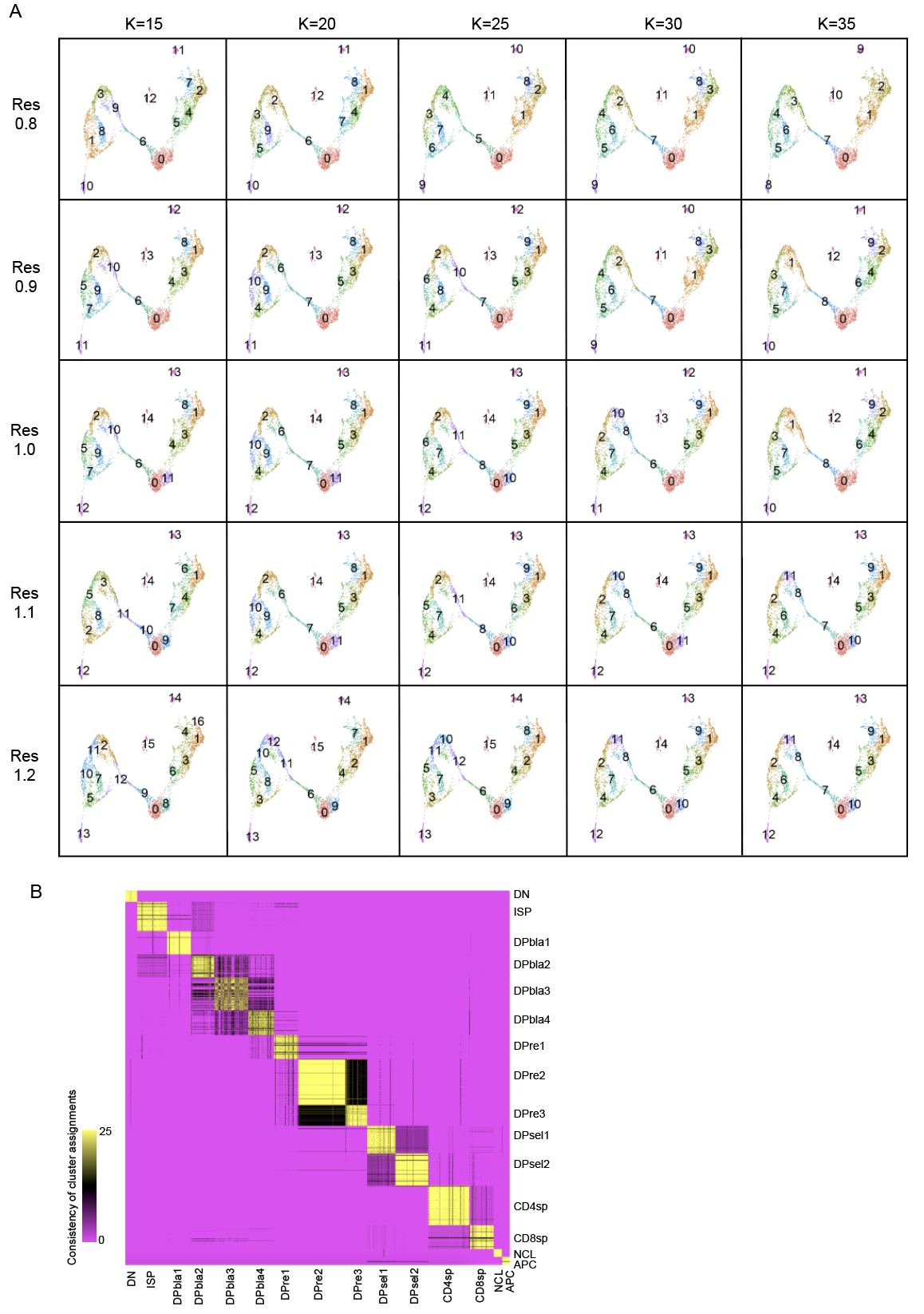


**Fig. S3 Robustness of thymocyte clustering**

**A:** Two-dimensional UMAP representation of integrated thymocytes, colored by the cluster identities. Five resolution parameters (0.8 - 1.2) and five nearest-neighbor numbers k (15 - 35) were used in Seurat analysis. **B**: Consensus matrix of results from 25 reclustering analyses using five resolution parameters (0.8 - 1.2) and five nearest-neighbor numbers k (15 - 35); entries indicate the number of analyses in which each pair of cells were assigned to the same cluster.


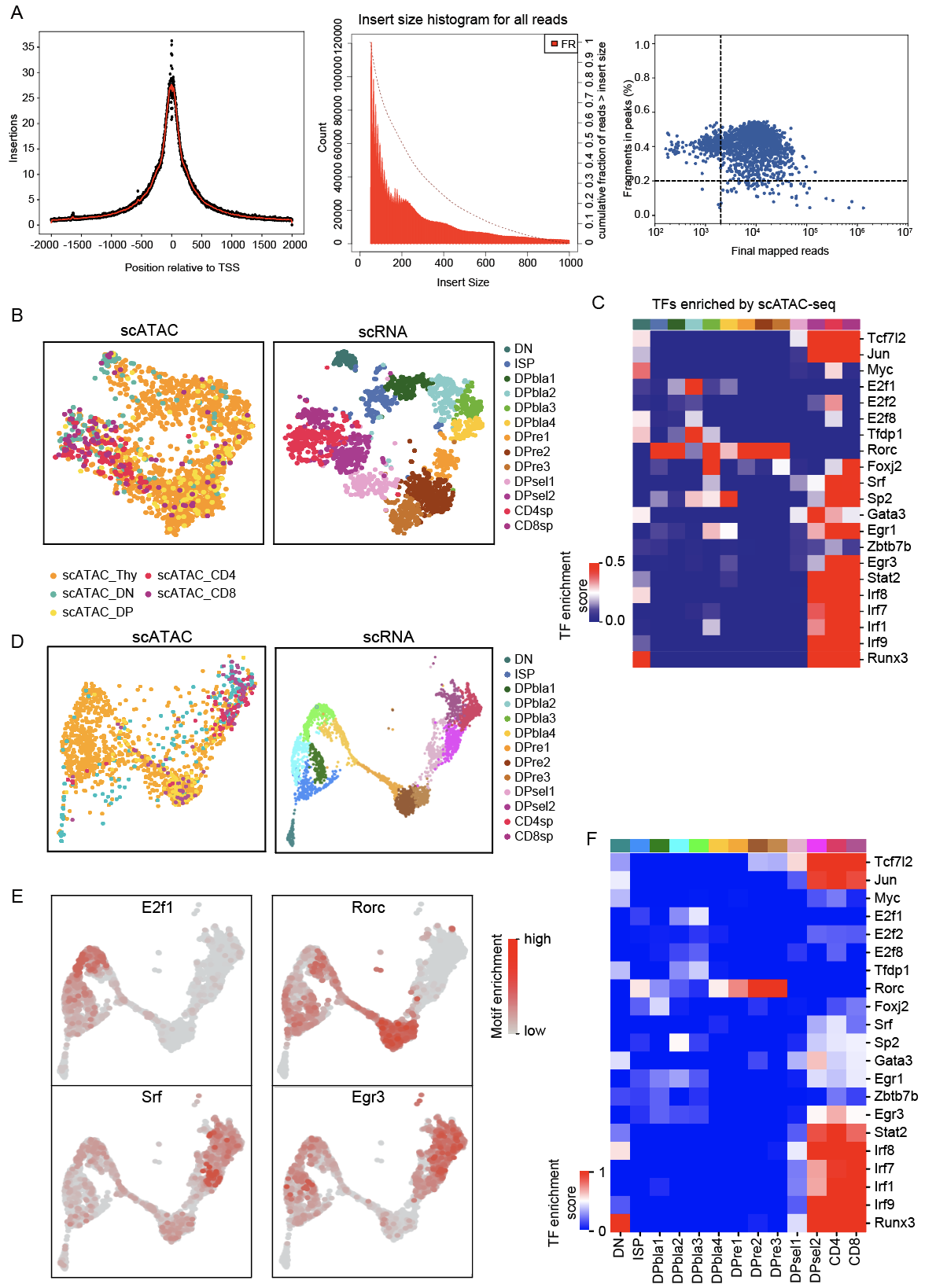


**Fig. S4 Single-cell ATAC-seq on mouse thymocytes**

**A:** Data quality of single cell ATAC-seq for thymocytes**. B:** scATAC-seq data were mapped onto scRNA-seq data (see Methods) and are displayed in a two-dimensional t-SNE plot. Sorted subpopulations were colored by source tags. Each dot represents one cell. **C:** Results of the TF enrichment in scATAC-seq. Clusters were labeled as in Fig. 1B. The color bar represents the average of the TF enrichment score for each stage. **D:** scATAC-seq data were mapped onto integrated scRNA-seq data (see Methods) and are displayed in a two-dimensional UMAP plot. Sorted subpopulations were colored by source tags. Each dot represents one cell. **E:** Enrichment of TFs (E2f1, Rorc, Srf and Egr3) during thymocyte development (see Methods) in scATAC data. And displayed in integrated data. The color bar represents the deviation in TFs. Each dot represents one cell. **F:** Results of the TF enrichment in scATAC-seq and displayed in integrated data. Clusters were labeled as in Fig. 1B. The color bar represents the average of the TF enrichment score for each stage.


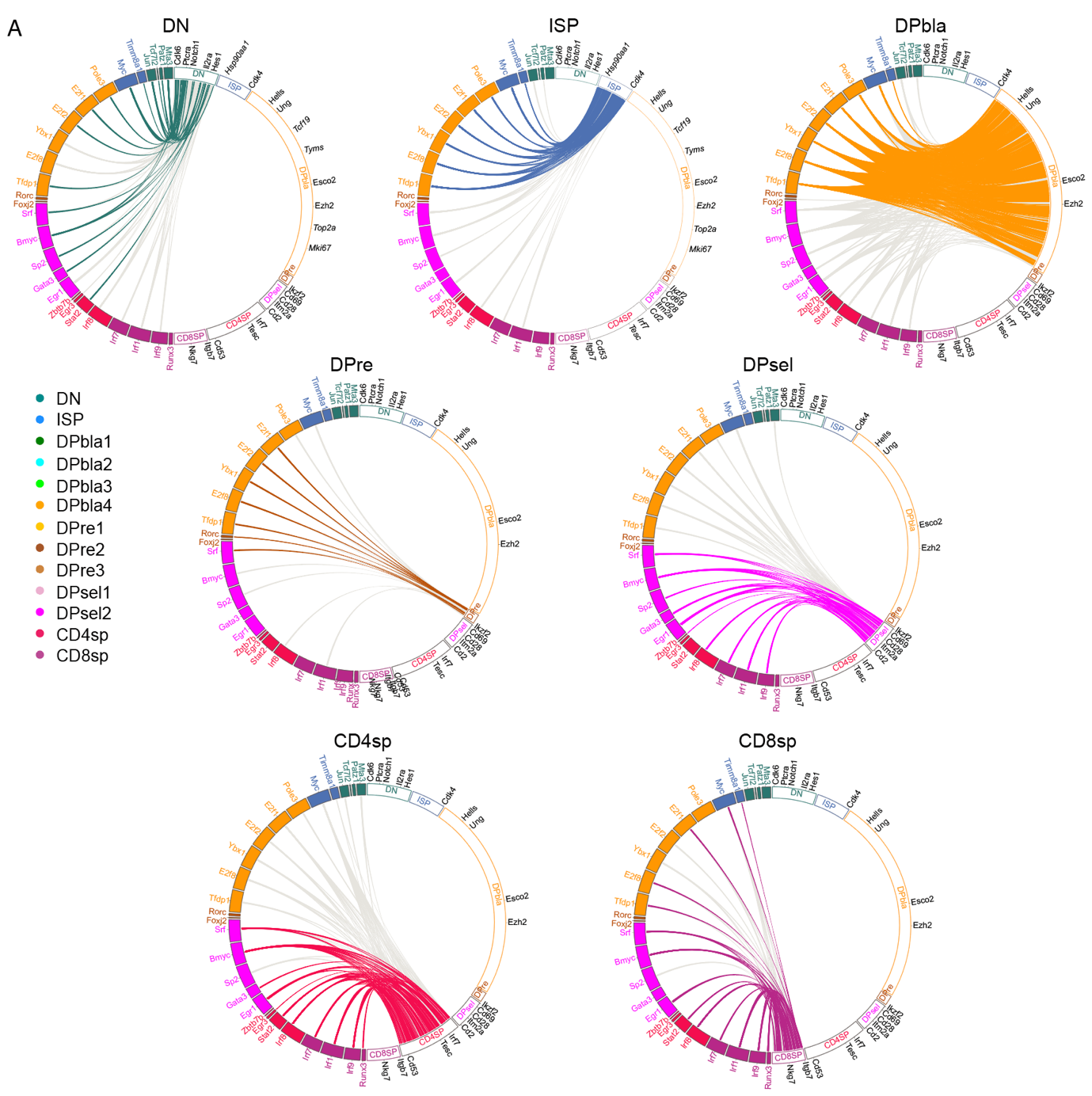


**Fig. S5 The transcription regulation network of each stage during thymocyte development**

**A:** Circos plot describes the regulation of genes by TFs. The left side shows the TFs enriched in each stage as in Fig.2B. On the right is the gene that is differentially expressed at each stage as in Fig.2A, some marker genes at each stage are marked outside the circle. The link from left to right indicates that the TFs regulate the corresponding genes. Gray link indicates that the correlation between TF and gene is less than 0.4.

**
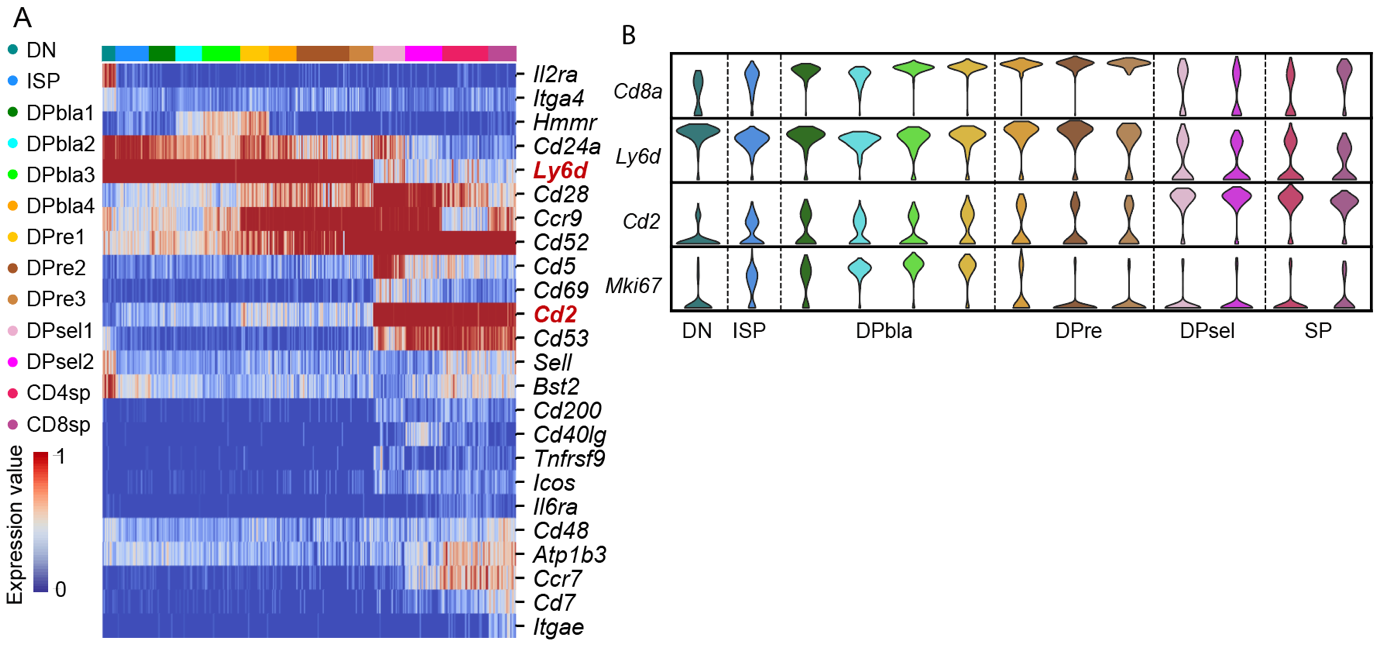
**

**Fig. S6 Ly6d and CD2 serve as new markers to gate DP subtypes in integrated data**.

**A:** Heat map of selected cell surface marker genes (color-coded by clusters). Columns denote cells; rows denote genes. **B**: Violin plots showing the normalized expression levels of selected marker genes that changed during the course of T cell development (*Cd8a, Ly6d, Cd2*, and *Mki67*).


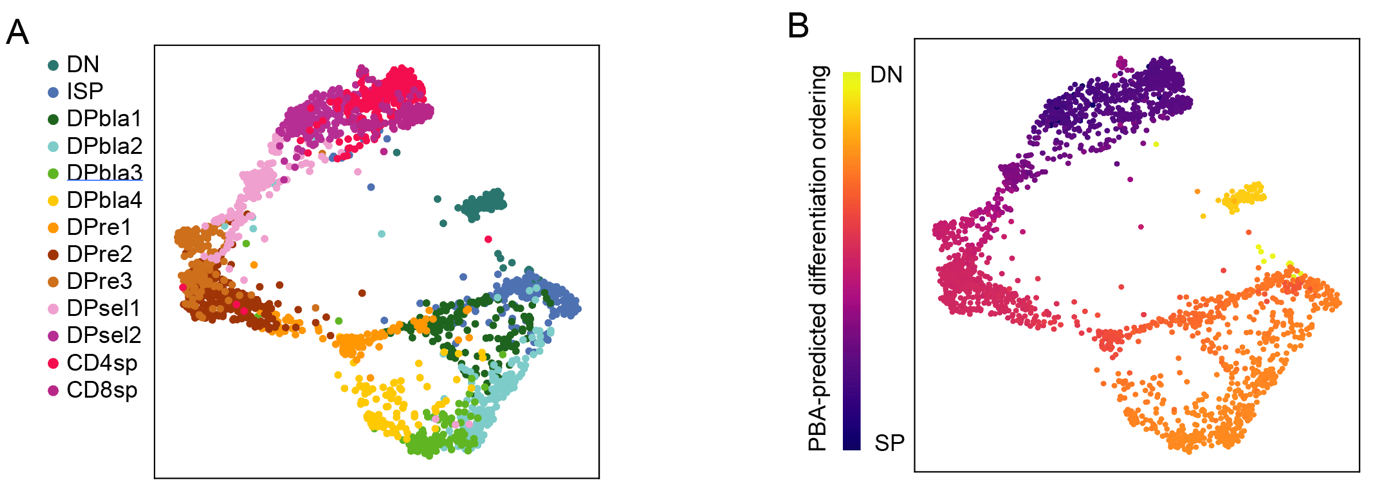


**Fig. S7 Single-cell transcriptome clustering and pseudo-time trajectory of thymocytes without cell cycle-related genes**

**A-B:** Two-dimensional representation of cells via t-SNE plots, colored by clusters (A) and the PBA-predicted differentiation order (B). Each dot represents one cell.


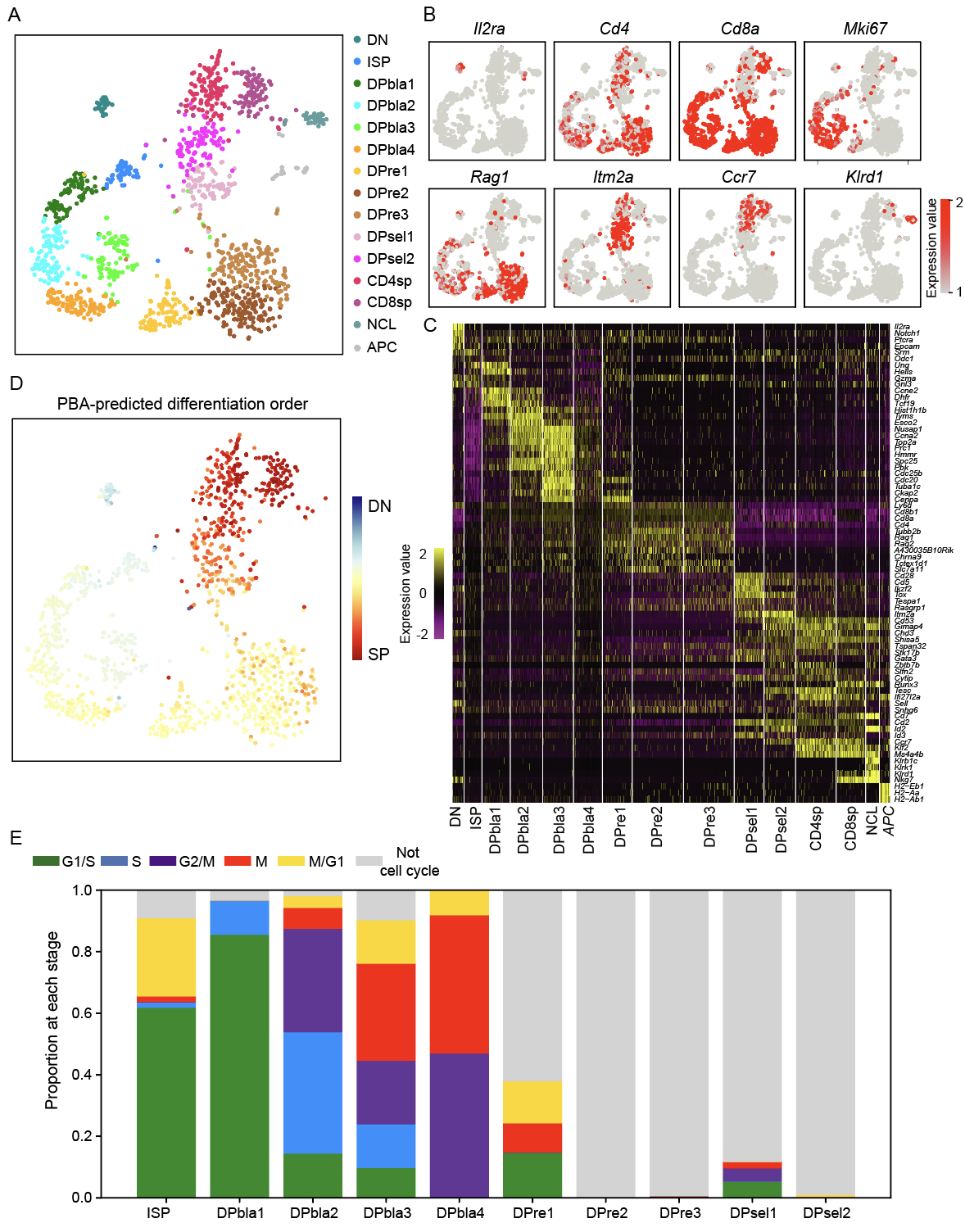


**Fig. S8 Single-cell transcriptome map of thymocytes from Tabula Muris dataset**

**A:** Two-dimensional representation of cells via a t-SNE plot, colored by the cluster identity. Each dot represents one cell. t-SNE was performed after quality control.

**B:** Marker genes projected on a t-SNE plot. The color bar represents the normalized expression value.

**C:** Heat map of cluster marker genes (color-coded by clusters), with exemplar genes labeled (right). Columns denote cells; rows denote genes.

**D:** Two-dimensional representation of cells via a t-SNE plot, colored by the PBA-predicted differentiation order. Each dot represents one cell.

**E:** The distribution of different cell cycle states in each DP thymocyte subpopulation. Cell cycle states (G1/S, S, G2/M, M, M/G1 and not cell cycle) were assigned the same way as in Fig. 4.

**
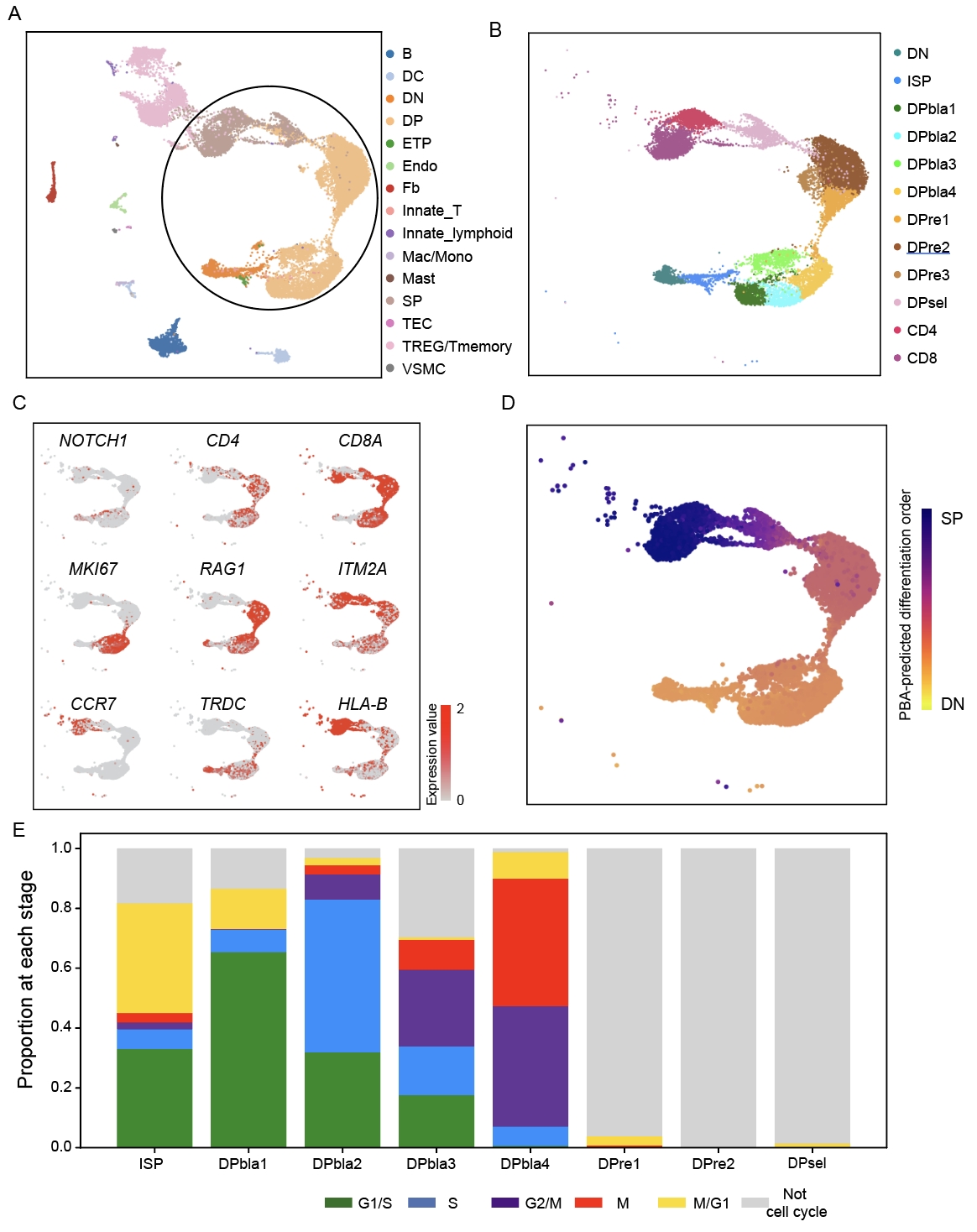
**

**Fig. S9 Single-cell transcriptome map of thymocytes from Human data**

**A:** Two-dimensional representation of cells via a UMAP plot, colored by the cluster identity. Each dot represents one cell. UMAP was performed after quality control. The label of the cell is obtained from the original article.

**B:** Two-dimensional representation of cells via a UMAP plot, colored by the cluster identity. Each dot represents one cell. UMAP was performed after quality control.

**C:** Marker genes projected on a UMAP plot. The color bar represents the normalized expression value.

**D:** Two-dimensional representation of cells via UMAP plot, colored by the PBA-predicted differentiation order. Each dot represents one cell.

**E:** The distribution of different cell cycle states in each DP thymocyte subpopulation. Cell cycle states (G1/S, S, G2/M, M, M/G1 and not cell cycle) were assigned the same way as in Fig. 4.


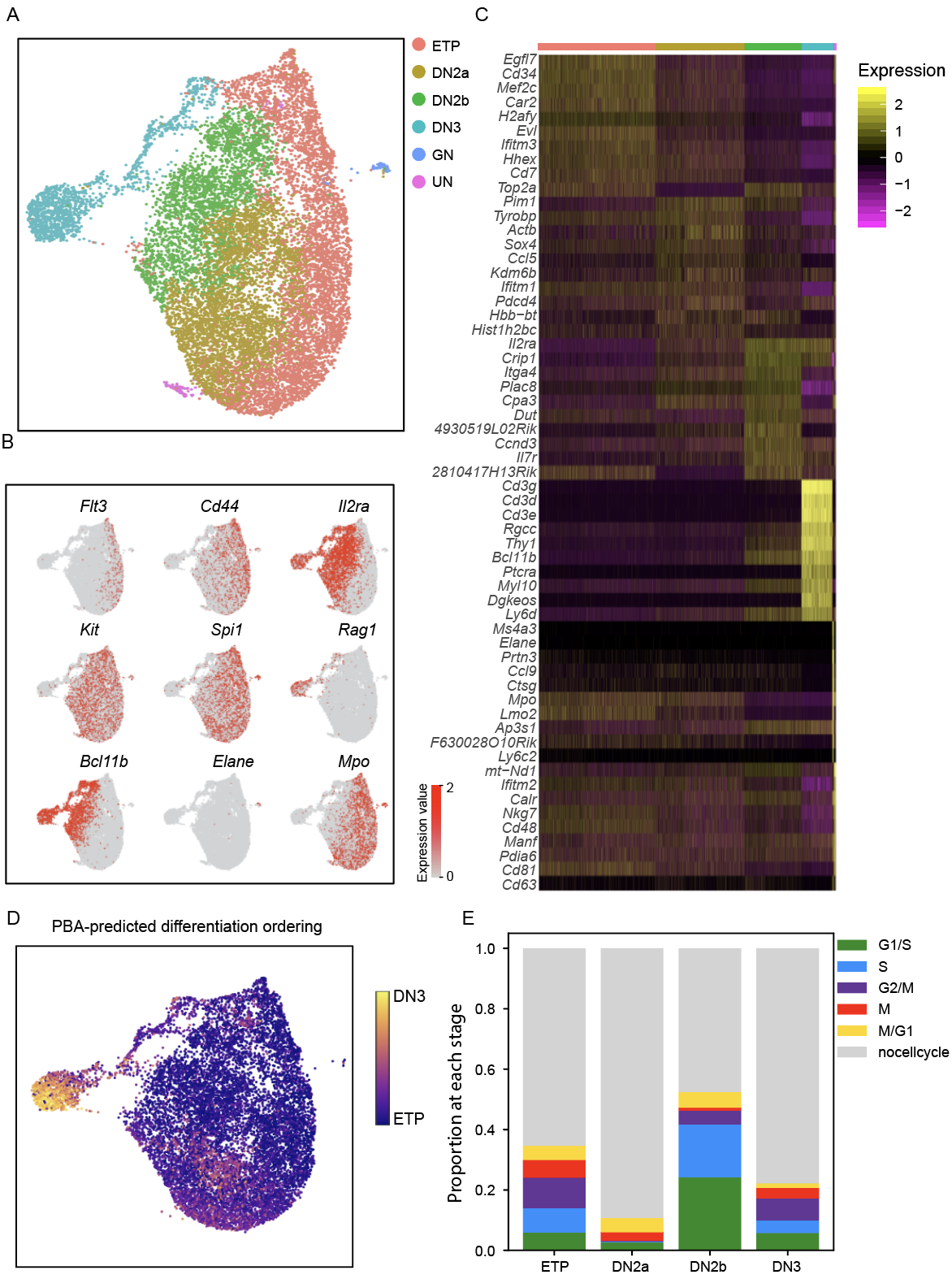


**Fig. S10 Single-cell transcriptome map of Early T Cell Development**

**A:** Two-dimensional representation of cells via a UMAP plot, colored by the cluster identity. Each dot represents one cell. UMAP was performed after quality control.

**B:** Marker genes projected on a UMAP plot. The color bar represents the normalized expression value.

**C:** Heat map of cluster marker genes (color-coded by clusters), with exemplar genes labeled (right). Columns denote cells; rows denote genes.

**D:** Two-dimensional representation of cells via a UMAP plot, colored by the PBA-predicted differentiation order. Each dot represents one cell.

**E:** The distribution of different cell cycle states in each thymocyte subpopulation. Cell cycle states (G1/S, S, G2/M, M, M/G1 and not cell cycle) were assigned the same way as in Fig. 4.


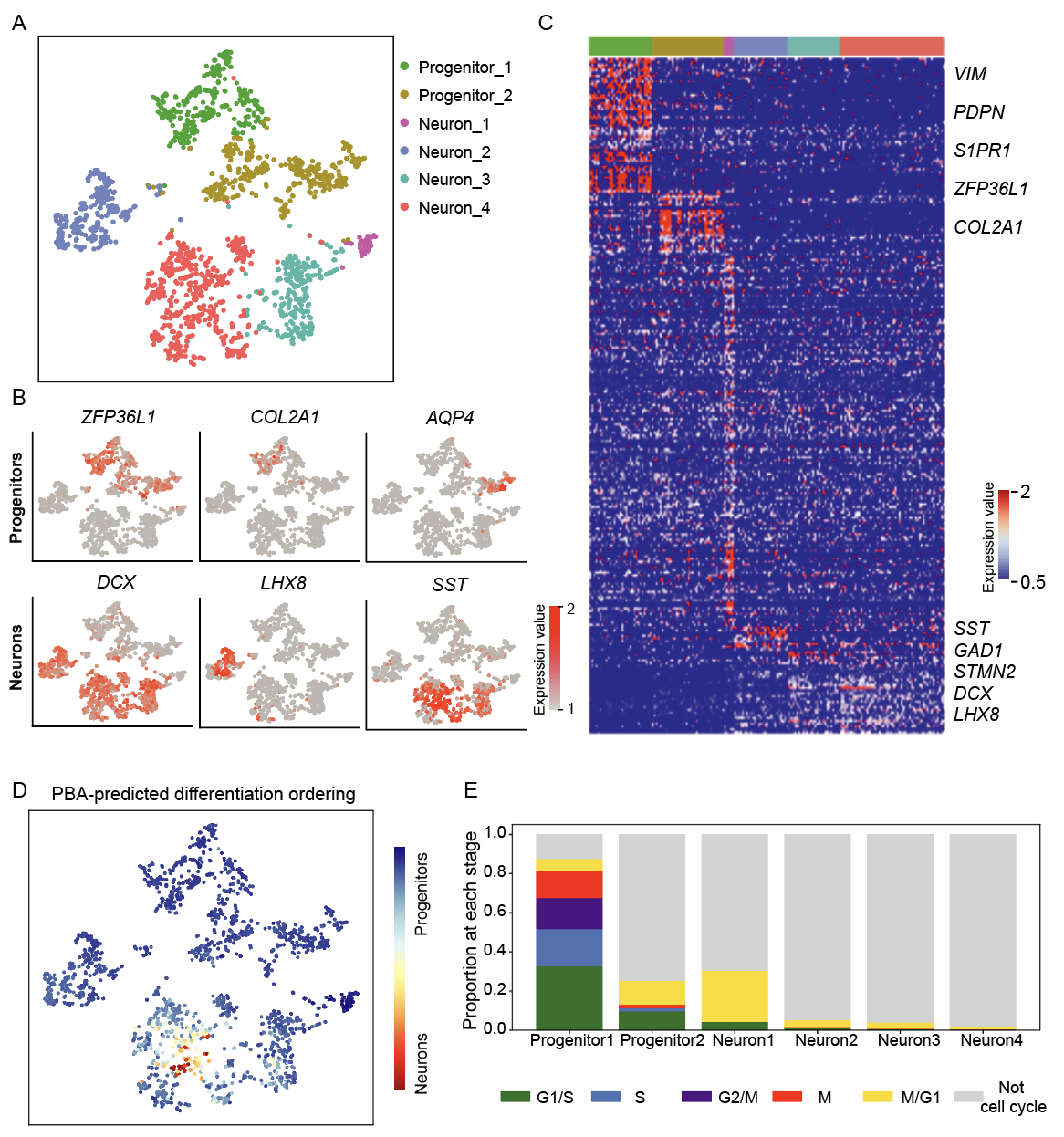


**Fig. S11 Single-cell transcriptome map of neuronal development**

**A:** Two-dimensional representation of cells via a t-SNE plot, colored by the cluster identity. Each dot represents one cell. t-SNE was performed after quality control.

**B:** Marker genes projected on a t-SNE plot. The color bar represents the normalized expression value.

**C:** Heat map of cluster marker genes (color-coded by clusters), with exemplar genes labeled (right). Columns denote cells, and rows denote genes.

**D:** Two-dimensional representation of cells via a t-SNE plot, colored by the PBA-predicted differentiation order. Each dot represents one cell.

**E:** The distribution of different cell cycle states in neuronal subpopulations. Cell cycle states (G1/S, S, G2/M, M, M/G1 and not cell cycle) were assigned the same way as in Fig. 4.


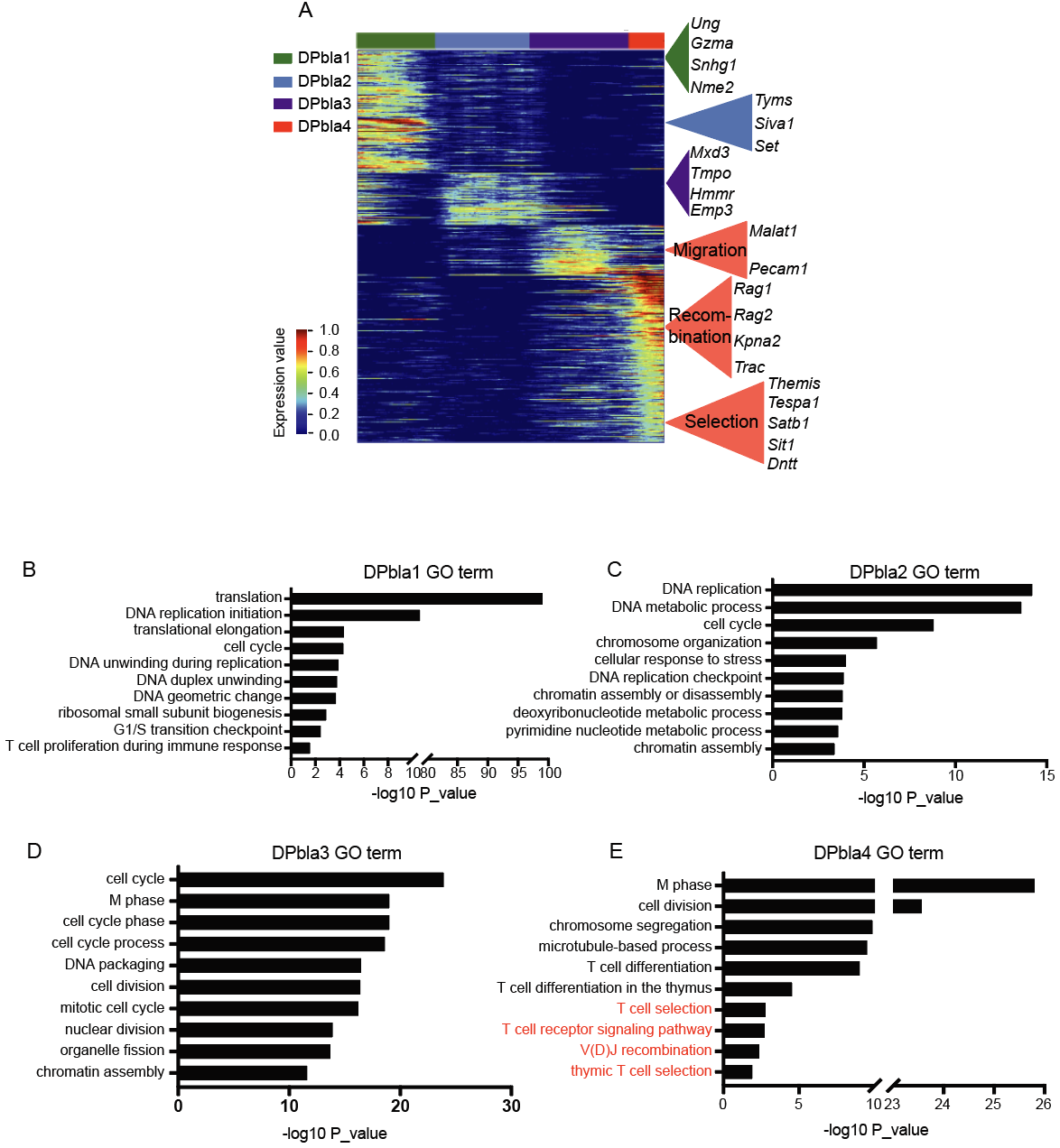


**Fig. S12 GO terms enriched in subgroups of DPbla stage.**

**A:** Heat map of cluster marker genes (color-coded by the DPbla cluster), with exemplar genes labeled (right). Columns denote cells, and rows denote genes.

**B-E:** Top 10 GO terms enriched in DPbla1 (B), DPbla2 (C), DPbla3 (D) and DPbla4 (E). Red indicates the GO term associated with rearrangement and selection. GO term enrichment analysis was performed with the Database for Annotation, Visualization, and Integrated Discovery (DAVID).


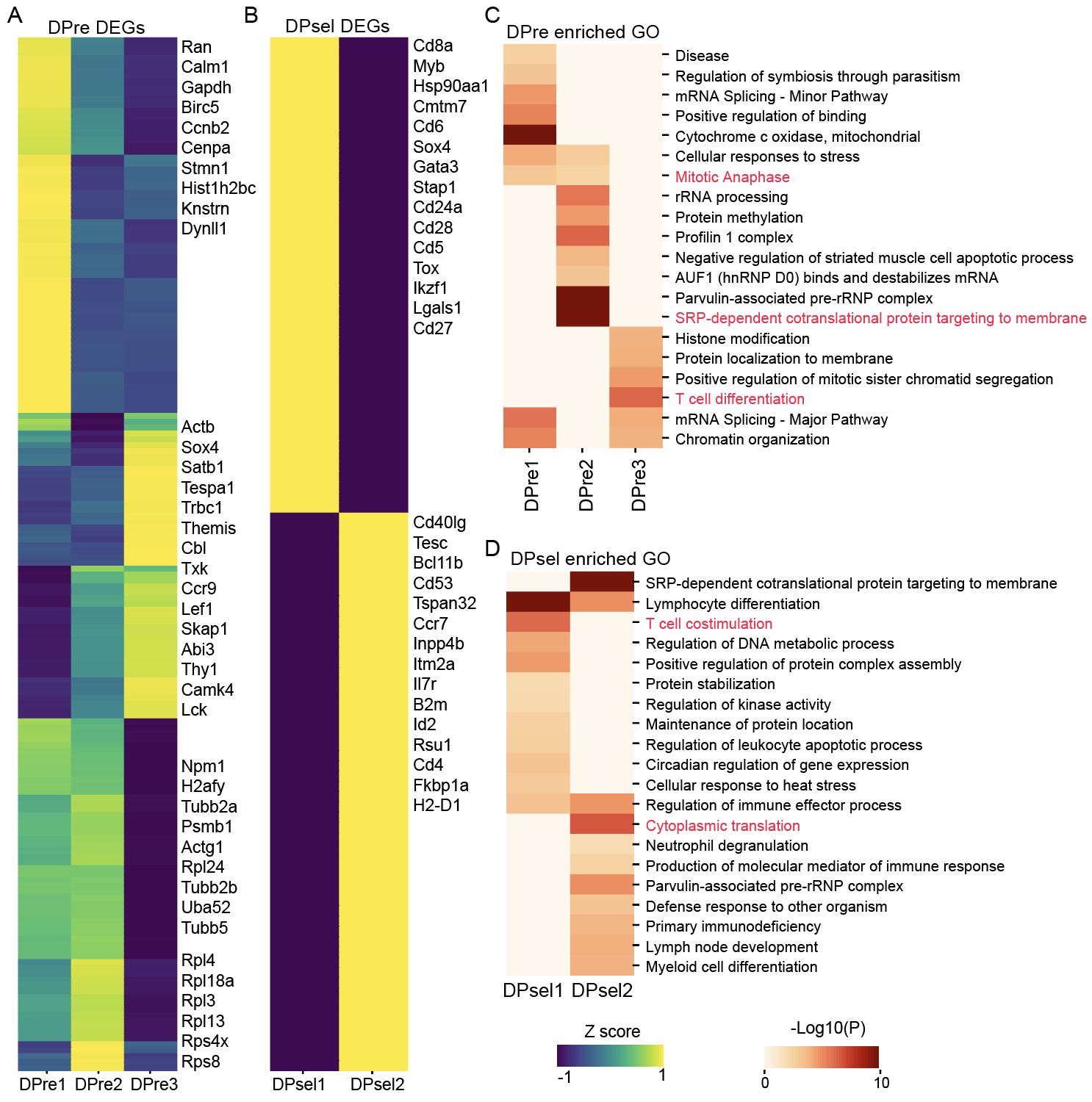


**Fig. S13 The difference in subgroups of DP stage.**

**A-B:** Heat map of stage marker genes of DPre (A) and DPsel (B), with exemplar genes labeled (right). Columns denote stages, and rows denote genes. **C-D:** GO terms enriched in subgroups of DPre (C) and DPsel (D). Red indicates the GO term associated with T cell development. GO term enrichment analysis was performed with Metascape.

**
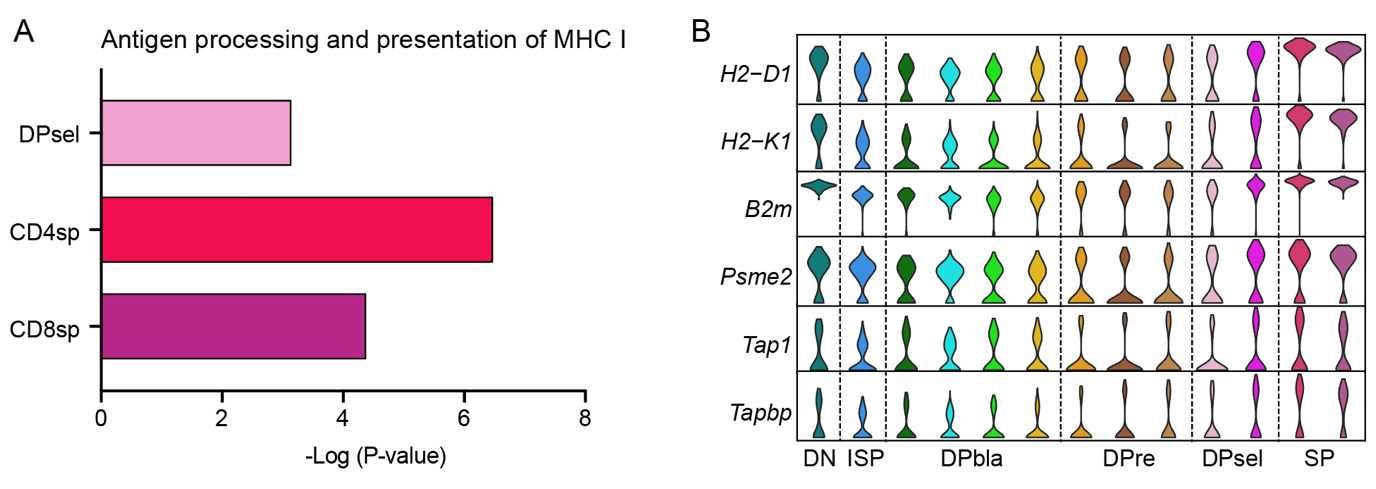
**

**Fig. S14 MHC-I antigen presentation occurred between thymocytes in integrated data**.

**A:** Top enriched GO terms in the marker genes of DPsels, CD4sps, and CD8sps. GO term enrichment analysis was performed with DAVID. **B:** Violin plots showing the normalized expression levels for selected marker genes that changed during the course of T cell differentiation (*H2-D1*, *H2-K1*, *B2m*, *Psme2*, *Tap1* and *Tapbp*).

**
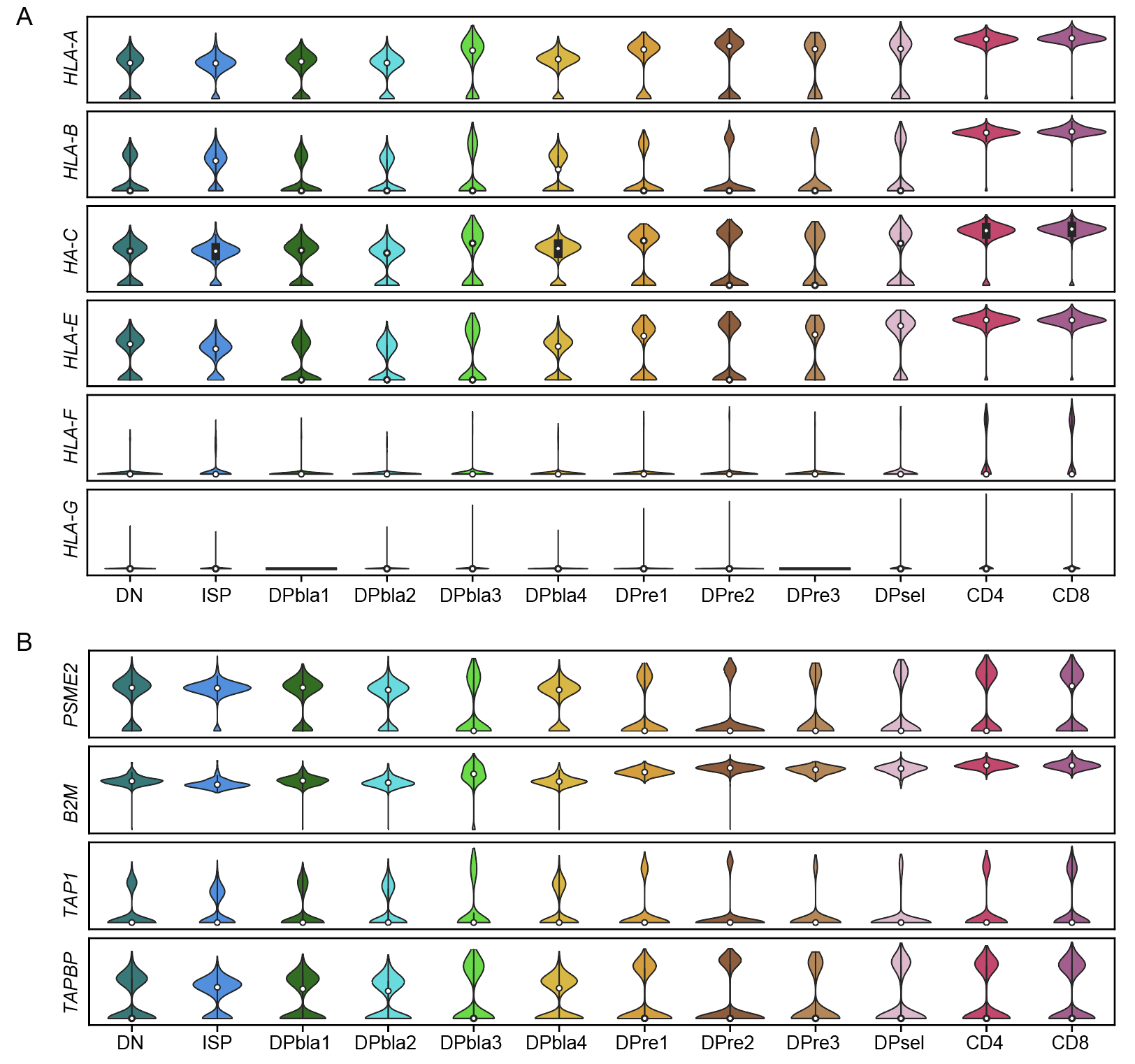
**

**Fig. S15 The expression of MHC-I associated genes in thymocytes.**

**A:** Violin plots showing the normalized expression levels of selected marker genes that changed during the course of Human T cell development (*HLA-A*, *HLA-B*, *HLA-C*, *HLA-E*, *HLA-F* and *HLA-G*).

**B:** Violin plots showing the normalized expression levels of selected marker genes that changed during the course of Human T cell development (*PSME2*, *B2M*, *TAP1* and *TAPBP*).


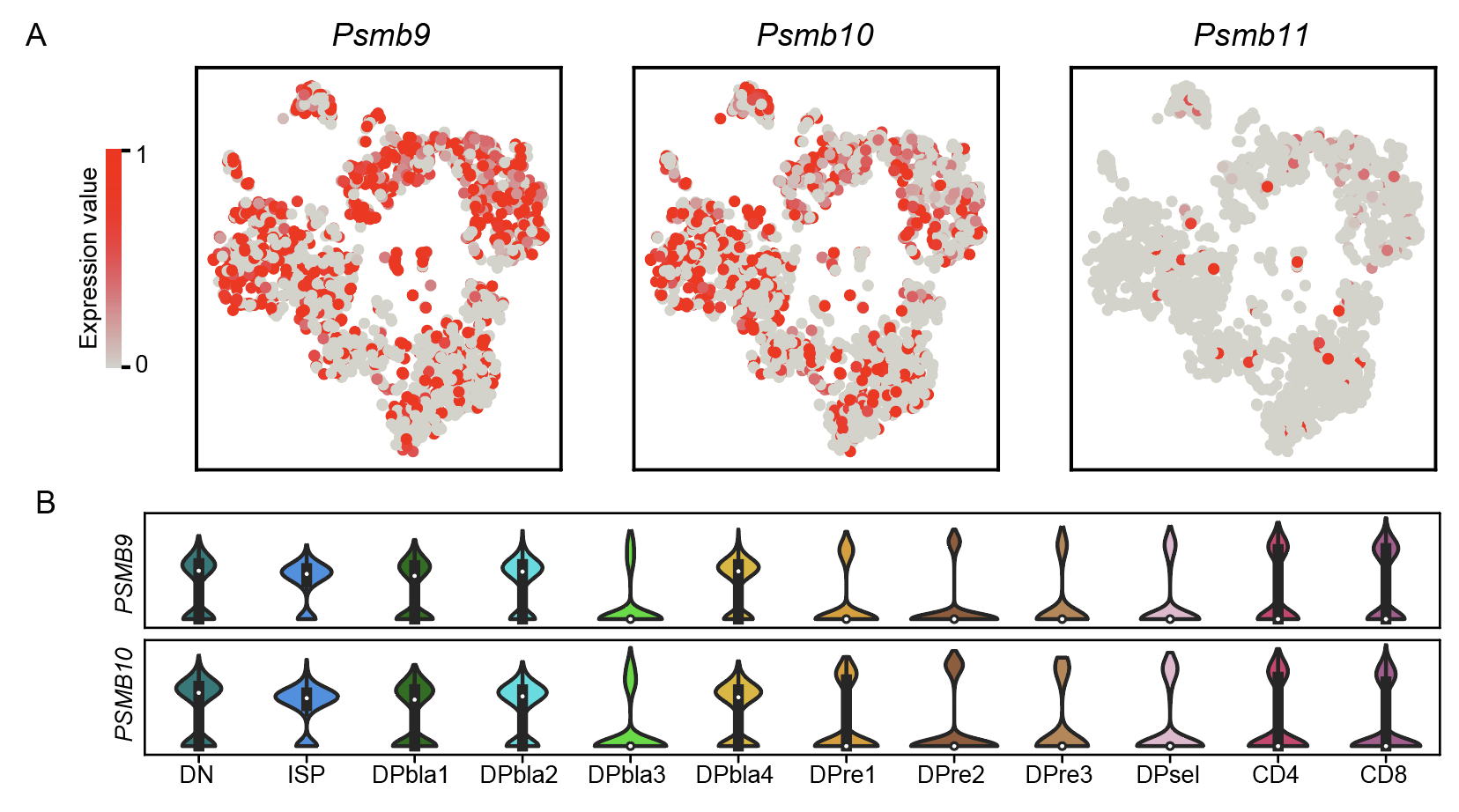


**Fig. S16 The expression of thymoproteasome subunits associated genes in thymocytes.**

**A:** *Psmb9*, *Psmb10* and *Psmb11* expression projected on t-SNE plots. The color bar represents the normalized expression value.

**B:** Violin plots showing the normalized expression levels of selected marker genes that changed during the course of Human T cell development (*PSMB9* and *PSMB10*).


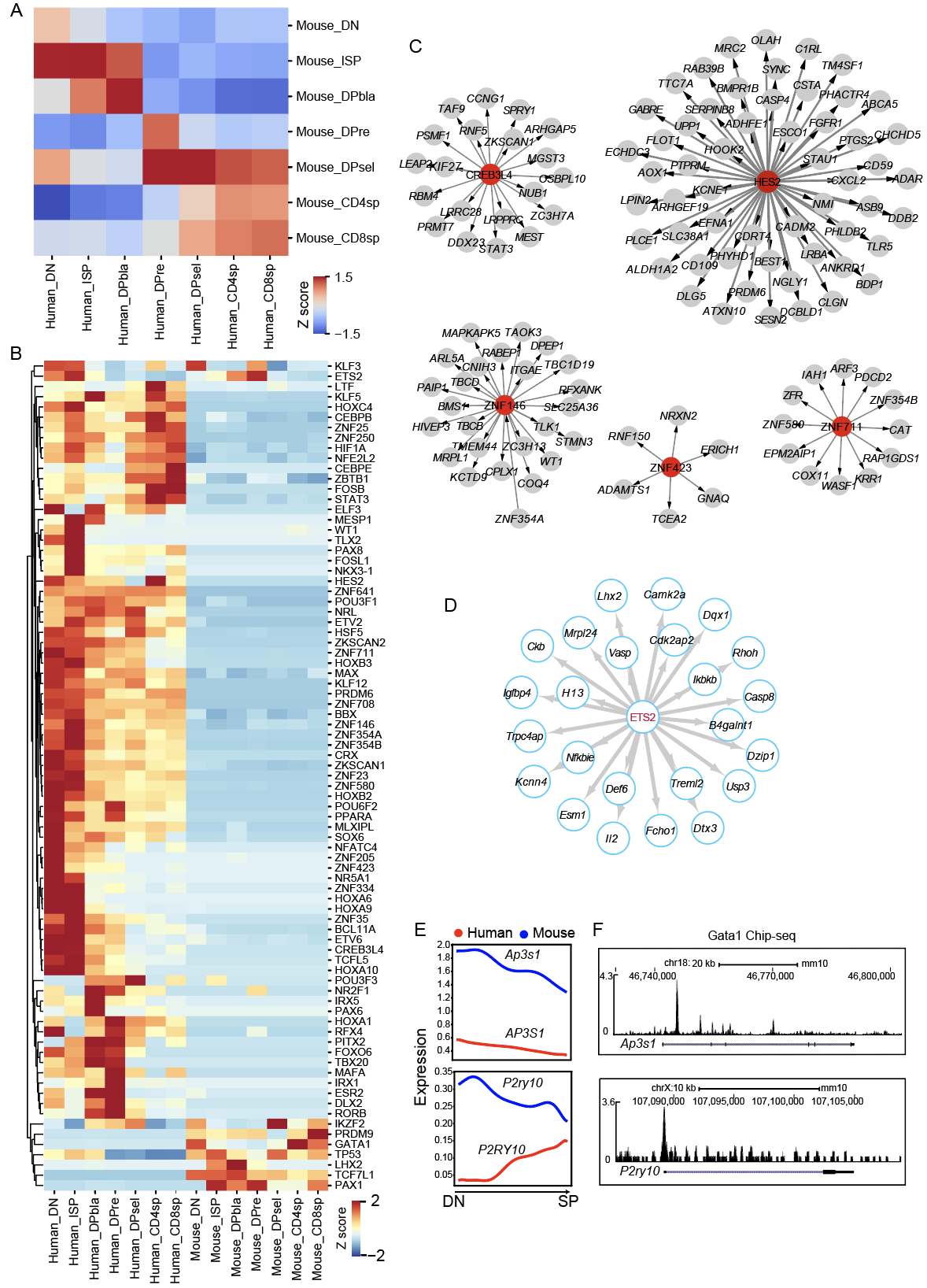


**Fig. S17. Transcriptional regulation differences between human and mouse thymocyte development**.

**A:** Pearson correlation between mouse data and human data. **B:** Heat map of selected TFs. Columns denote stages; rows denote TFs. **C-D:** The transcriptional regulatory network of CREB3L4, HES2, ZNF146, ZNF423, ZNF711 (C) and ETS2 (D). Each node is a gene, the red one is TF. Shown are genes differentially expressed between species in Fig.6B. **E:** Expression of representative genes *AP3S1* and *P2RY10* in the development of T cell. Value is the smoothed gene expression value. **F:** Gata1 ChIP-seq profiles of *Ap3s1* and *P2ry10* gene loci. The Gata1 ChIP-seq data is from Leukemia cells and ES (embryonic stem) cell derived erythroid progenitors. ChIP-seq data were obtained from the Cistrome Data Browser.
